# An Aerial–Wall Robotic Insect That Can Land, Climb, and Take Off from Vertical Surfaces

**DOI:** 10.34133/research.0144

**Published:** 2023-05-10

**Authors:** Qian Li, Haoze Li, Huan Shen, Yangguang Yu, Haoran He, Xincheng Feng, Yi Sun, Zhiyuan Mao, Guangming Chen, Zongjun Tian, Lida Shen, Xiangming Zheng, Aihong Ji

**Affiliations:** ^1^ College of Mechanical and Electrical Engineering, Nanjing University of Aeronautics and Astronautics, Nanjing, China.; ^2^ College of Aerospace Engineering, Nanjing University of Aeronautics and Astronautics, Nanjing, China.; ^3^ State Key Laboratory of Mechanics and Control for Aerospace Structures, Nanjing University of Aeronautics and Astronautics, Nanjing, China.

## Abstract

Insects that can perform flapping-wing flight, climb on a wall, and switch smoothly between the 2 locomotion regimes provide us with excellent biomimetic models. However, very few biomimetic robots can perform complex locomotion tasks that combine the 2 abilities of climbing and flying. Here, we describe an aerial–wall amphibious robot that is self-contained for flying and climbing, and that can seamlessly move between the air and wall. It adopts a flapping/rotor hybrid power layout, which realizes not only efficient and controllable flight in the air but also attachment to, and climbing on, the vertical wall through a synergistic combination of the aerodynamic negative pressure adsorption of the rotor power and a climbing mechanism with bionic adhesion performance. On the basis of the attachment mechanism of insect foot pads, the prepared biomimetic adhesive materials of the robot can be applied to various types of wall surfaces to achieve stable climbing. The longitudinal axis layout design of the rotor dynamics and control strategy realize a unique cross-domain movement during the flying–climbing transition, which has important implications in understanding the takeoff and landing of insects. Moreover, it enables the robot to cross the air–wall boundary in 0.4 s (landing), and cross the wall–air boundary in 0.7 s (taking off). The aerial–wall amphibious robot expands the working space of traditional flying and climbing robots, which can pave the way for future robots that can perform autonomous visual monitoring, human search and rescue, and tracking tasks in complex air–wall environments.

## Introduction

After hundreds of millions of years of evolution, insects in nature not only possess amazing flying skills [1–4] but also can climb and adhere to surfaces made of various materials [5–7]. The complex flapping movement of insects during flight is the key to generating high lift and maintaining efficient and agile flight. On the one hand, unsteady high lift is obtained from the flow characteristics generated by flapping insects [8–10]. On the other hand, insects can easily complete actions with multiple degrees of freedom such as roll, pitch, yaw, and hover by adjusting their posture, and they can control their flight stability even in harsh environments or when burdened with heavy loads [1,2,11,12]. The crawling ability of insects on the surface of materials with different roughness is closely related to the soles of their feet. On rough surfaces, insects use claws at the ends of their toes (or anterior tarsi) to attach themselves [7]. By contrast, on relatively smooth surfaces, insects use smooth pads or setae pads [13]. Research on the synergistic adhesion behavior of the hooks and setae has revealed that the adhesion force generated by a structure consisting of both the hook and setae is larger than the sum of the 2 structures alone, which reflects their synergistic effect. This synergy greatly expands the range of surfaces to which insects can attach [14]. Inspired by the locomotion of insects that take off, flap their wings, and land on a vertical wall to climb it, we implemented these movements on an aerial–wall amphibious robot.

### Various wall-climbing robots and their limitations

In past decades, various forms of wall-climbing robots have been studied and developed, and their main motion modes can be divided into (a) clawed wall-climbing robots for rough walls that use foot claws [15–17] or wheel claws [18], and (b) adhesive-pad wall-climbing robots for smooth walls that use climbing adhesion [19,20]. In particular, the adhesive-pad robot has the ability to perform flexible and stable climbing and jumping movements on a variety of surfaces with different roughness like insects because of its adhesive force, which is similar to that of an insect foot pad. Some advanced adhesive-pad wall-climbing robots can even perform difficult tasks, such as searching inside pipelines in extreme environments, inspecting the exterior of oil tanks, or cleaning glass surfaces [18,21]. These robots are able to climb on the external surfaces of buildings made of materials such as plaster and bricks using tiny spines similar to those of insects [15]. Smooth vertical surfaces such as windows and interior walls are climbed using suction [22,23], magnetic force [21], and pressure sensitive adhesives (PSAs) such as tape [18]. Some adhesive materials such as PSAs have high adhesion on smooth surfaces, but are prone to fouling and require relatively high forces to adhere and peel. All of these solutions are successful, but their uses are limited. The ultimate goal of wall-climbing robots is to achieve insect-like maneuverability and stability in secret reconnaissance and disaster search and rescue on challenging surfaces or complex indoor environments.

The safe movement of wall-climbing robots is hindered by unfavorable wall conditions and various external conditions such as the weather. This limits their practical use to wall-friendly indoor environments. This limitation often means they are a highly engineered system, as can be seen in the case of some window-cleaning robots. In addition, the application of wall-climbing robots is limited to movements occurring on low walls because it is challenging for them to access a higher position.

### Various aerial robots and their limitations

These difficulties can be overcome by aerial robots, which can carry loads and fly away from any area. Fixed-wing [24,25] and rotor-wing vehicles [26–28] have been extensively studied. In addition, biomimetic flapping-wing flight, which has emerged in recent years, can realize the back-and-forth pronation, supination, and a flapping movement similar to insect wings or hummingbird wings through an appropriate driving mechanism combined with flexible membranes. A flapping wing can also generate enough thrust when there is no forward flight speed to realize hovering flight like insects or hummingbirds [4,29–32]. However, these robots also have their limitations, which include high energy consumption, short flight and operation time, and limited load capacity. In addition, it is more difficult for aerial robots to physically interact with the environment or other robots than it is for wall robots because they need to remain stable in the air, which makes it difficult for them to interact with external physical quantities through aerial maneuvering.

### Advantages and research status of amphibious robots

Robots with the ability to move in various mediums have advantages over robots that move only in a single medium. Such robots have multiple locomotion modes and move through challenging environments by appropriately switching among these modes. For example, when inspecting the distal surface of buildings, dams, and other large structures, the robot can fly to the surface and stay there to deal with the harsh environment and weather. After the conditions improve or the task has been completed, the robot can detach from the wall and return to the air. However, the realization of such cross-medium robots has, in practice, been a challenge.

Initial studies focused on the development of land–aquatic amphibious robots [33,34], but recently, aerial–aquatic amphibious robots have been developed [35]. Plenty of robots have demonstrated the ability to move between air and land. A hexapod robot with flapping wings was used to explore the origin of bird flight, but it still cannot fly [36]. In recent years, LEO (a multimodal locomotion robot called LEONARDO, which is an acronym of LEgs ONboARD drOne) has bridged the gap between the 2 locomotion modes of flying and walking using the synchronous control of rotors and one pair of multiple articular legs, demonstrating adaptability on complex terrain [37].

The design of an aerial–wall amphibious robot is more closely related to the insects’ habits than the design of an air–land amphibious robot, which is an impressive accomplishment. In the aftermath of an earthquake or other disaster, walls tend to be relatively unobstructed, whereas the horizontal ground may be littered with debris. Staying a few meters above the ground provides a relatively safe position away from ground traffic, allowing the aircraft to take off without expending a lot of energy. In addition, landing on a vertical wall tends to be a fast process, and hence disturbances such as the wind have less impact than they do on hovering or flying slowly. Because of the demand of special applications, expanding the capability of an aircraft to climb on a wall has also become an important direction and popular topic in the research of bionic robots in recent years. For example, when an aircraft flies toward a wall, a pitch maneuver is triggered. The claws on the aircraft are attached to the pit on the surface, and the success rate of this type of vertical landing on a building wall is approximately 80% [38]. In [39], a vehicle was developed that could climb a prepared surface and then detach from the surface and enter a glide trajectory. A quadrotor equipped with a climbing mechanism realizes the ability to go from flying to landing on the wall, climbing the wall, and then taking off from the wall and returning to flight [40]. Thus far, no robot has been developed that can fly in the air and climb on vertical walls using insects as biomimetic objects, and no similar concept of robotic insect has been proposed.

### Significance and advantages of this research

Cross-domain movement between a wall and the air is a common form of movement for flying creatures such as insects, bats, and birds. Insects in particular fly to feed, avoid predators, or move to a more comfortable position. Even so, their climbing performance is also outstanding among animals. They not only can move quickly on the ground, a ceiling, or a vertical wall but also can attach themselves to branches.

**Fig. 1.**
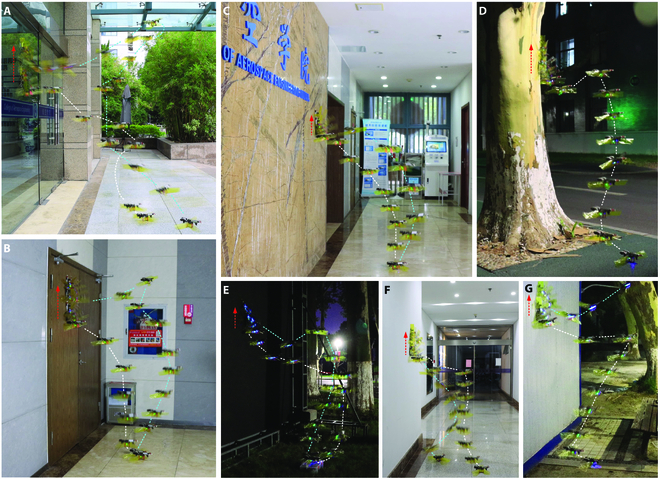
The robot landing, climbing, and taking off on a variety of vertical walls. (A) Glass door surface. (B) Wooden door surface. (C) Marble wall surface. (D) Tree surface. (E) Soft spray cloth surface. (F) Lime wall surface. (G) Painted iron sheet surface. The white dotted lines indicate the path of the robot taking off from the ground and flying toward the wall. The cyan dotted lines represent the path of the robot taking off from the wall and moving away or flying toward the ground, and the red dotted lines with arrows indicate the path of the robot climbing on the vertical wall.

Aerial–wall amphibious robots aim to bridge the gap between 2 completely different areas of aerial motion and wall-climbing motion that are not usually intertwined in existing robotic systems. Aerial–wall amphibious robots can focus on the multifunctional tasks that need to be accomplished in places that are difficult for wall-climbing robots to reach. In contrast to wall-climbing robots, aerial–wall amphibious robots can use the transition between climbing and flying modes to overcome any obstacle and can use flight mode to easily reach higher positions. In addition, while an aerial robot can hover over the target, its wall-climbing motion can also be used to approach the target for closer inspection. By taking advantage of its unique layout design and control strategy, the aerial–wall robot has successfully demonstrated its ability to land, climb, and take off from a variety of complex vertical walls (Fig. 1). This series of steps is very common in nature, for instance, when a fly arrives at a location after flying some distance, lands on a wall, climbs to a place where it feels comfortable, and then flies to the next place.

Compared with other schemes, the aerial–wall robot has the following advantages: (a) The aerial–wall robot integrates the advantages of flapping-wing and rotor-wing power through the layout of the flapping/rotor hybrid power. It has advantages in dynamic efficiency, attitude control, structural dimension, wall climbing, aerodynamic characteristics, bionic appearance, and other aspects. (b) The aerial–wall robot can realize any smooth transition between flying and climbing mode through a unique transition scheme. (c) Using the negative pressure of the rotor to enhance the adhesion climbing mechanism, the aerial–wall robot can achieve stable adhesion and fast climbing on the wall, and has strong adaptability to a variety of roughness on a vertical wall. The aerial–wall amphibious robot truly combines wall climbing with air flying movement, extending the movement environment, working space, and mission duration of traditional aircraft.

## Results

### Flapping/rotor hybrid power layout

**Fig. 2.**
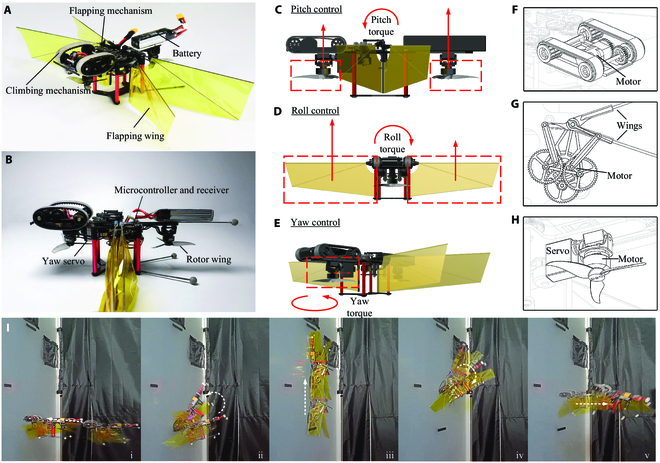
Details of the components and tracking marker positions of the robot. The insect-inspired free-flying robotic platform is controlled through its 2 pairs of independently flapping wings and rotors. (A and B) Description of the robot’s components. (C to E) Wing actuation and aerodynamic forces and torques during pitch control (C), roll control (D), and yaw control (E). Red arrows show the thrust and torques after control actuation. (F to H) Details of the robot design: (F) the climbing mechanism for wall climbing control, (G) the flapping mechanism (of the right wing pair) used for roll torque control, and (H) the rotor adjustment mechanism for yaw torque control. (I) High-speed camera frames capturing the robot during getting closer (i), landing (ii), climbing (iii), taking off (iv), and leaving away from the vertical surfaces (v), from Movie S1.

The aerial–wall robot adopts a hybrid layout with flapping wings and rotors, as shown in Fig. 2. The 2 flapping-wing power groups are symmetrical on the horizontal axis, and the 2 rotor power groups are respectively located at the head and tail of the longitudinal axis of the fuselage. The 4 groups of power are arranged in a "+" shape. The head rotor power group adopts the design of vector power, which can be driven by a vector servo to perform the deflection.

This hybrid power arrangement of flapping wings and rotors can provide stable attitude control for the aerial–wall robot. The pitch moment is generated by the differential rotation of the head and tail rotor power group, which controls the pitch and longitudinal motion of the robot. The differential flapping of the left and right flapping wings generates roll moment, which controls the roll and lateral motion of the robot. The vector deflection servo drives the head rotor power group to deflect and generate the yaw deflection moment, which provides the yaw motion control for the robot.

Compared with the conventional flapping-wing aircraft, the design scheme of hybrid flapping/rotor power layout has many advantages: (a) In terms of power efficiency, the small rotor improves the ultimate thrust, while the flapping wing improves ultimate efficiency, and the efficiency of the combined flapping and rotor power is 0.031 N/W, as shown in Fig. S1. (b) With respect to attitude control, compared with wing root or vector control, longitudinally distributed rotors can provide larger and more direct pitch and yaw moments over a larger range of pitch angle, and the designed fuselage structure is more concise and reliable. (c) In terms of structure size, compared with a "+" 4 flapping-wing aircraft, the longitudinal distribution of small rotors reduces the length along the longitudinal axis, which avoids interference between the longitudinal flapping wing and the wall, and is more conducive to the longitudinal flip action of the robot after it touches the wall. (d) In terms of wall climbing, a flapping wing relies on the flapping mechanism to generate aerodynamic force, but rotors do not produce strong vibrations, which can provide more stable negative pressure for the climbing mechanism and resisting the overturning moment caused by gravity. The third and fourth advantages are detailed in the next section.

The flapping-wing power group also brings important advantages to the robot in terms of aerodynamics and bionics. For example, when the robot flies forward at high speed, lift force is generated by the airfoil of the flapping wing similar to a fixed wing under an incoming flow, which can effectively improve flight efficiency. The flapping-wing power group has a vivid bionic appearance and flapping morphology, and when coordinated with a small transparent rotor, gives the robot a stronger visual charm and makes it less visible.

### Flying–climbing–flying transition scheme

The climbing power group of the aerial–wall robot is located on the dorsal side of the fuselage with the adhesion surface facing upwards, which is more simple and direct for the flying–climbing–flying transition, as shown in Fig. 2A. To prepare for the flying–climbing transition, the robot reduces the flying speed and slowly approaches the wall. When the adhesion pad at the front of the climbing power group contacts the wall, the flight control maintains the Stabilize mode, which keeps the pitch and roll attitude self-calibrated, and the desired pitch angle gradually decreases. The robot rotates around the contact point at the front of the climbing mechanism, and the tail gradually rises. Once the flight control determines that the actual pitch angle is tilted downward at the critical angle −60°, the Stabilize mode is automatically converted to the Acro mode, in the remote control (RC) stick is used to control the angular velocity of the robot in each axis. In addition, the flapping-wing power group is automatically closed. After the pitch angle exceeds the critical angle, the expected pitch moment gradually decreases until the adhesive pad of the climbing power group fully touches the wall. The tail battery leans against the wall, and the flying–climbing transition phase is finished, as shown in Fig. 2I(i) to (iii).

In the latter part of the flying–climbing transition and during the climbing stage, the flapping-wing power group is kept closed. The rotor power direction is toward the wall, and the rotor thrust is increased or decreased synchronously to adjust the negative pressure and asynchronously to adjust the anti-overturning moment. The servo deflection can change the pressure of the belts on both sides to control the climbing direction. In the climbing stage, the robot is closely attached to the wall relatively stably, and continues to operate in Acro mode. The main function of the thrust of the rotor power group in the head of the robot is to provide pressure for the adhesion pad of the climbing power group to enhance the adsorption and friction during climbing. The main function of the thrust of the rotor power group in the tail is to resist the overall overturning moment of the robot and press the tail stably on the wall, as shown in Fig. 2I(iii).

In preparation for the climbing–flying transition, the thrust of the robot’s head rotor power group is kept almost constant to keep the front of the climbing power group in persistent contact with the wall. The power of the tail rotor is gradually decreased, and the robot will be affected by the overturning moment. As a result, the tail will naturally detach from the wall and fall. When the flight control determines that the actual pitch angle is restored to the critical angle of −60°, the flapping-wing power group is automatically activated and Stabilize mode is automatically actuated. In the subsequent process of leaving the wall, the pitch angle and angular velocity of the robot should be accurately controlled. As gravity acts on the mass of the robot, the falling speed and pitch angular velocity of the robot will gradually increase. If the robot is left to accelerate naturally, it will lose control and fall rapidly. Therefore, it is necessary to enhance the power of the tail rotor in advance and coordinate the head rotor to reduce the falling speed and pitch angular velocity to almost zero before the robot reaches the horizontal attitude so that the robot enters a stable and controllable hovering flight. At this moment, the climbing–flying transition ends and the robot re-enters the flight phase, as shown in Fig. 2I(iii) to (v).

### Negative pressure of the rotor to enhance adhesion during climbing

**Fig. 3.**
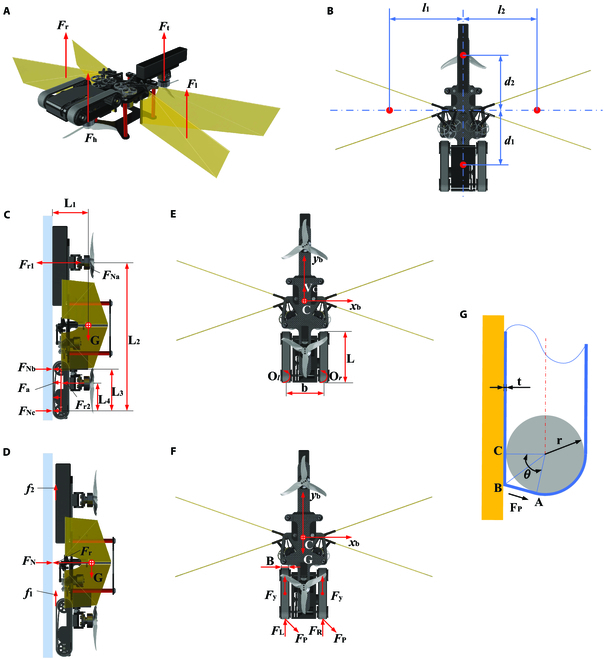
Kinematic and dynamic models of the robot. (A) Flight dynamical model. *F*_h_, *F*_t_, *F*_l_, and *F*_r_ are the thrust forces generated by the head rotor, tail rotor, left flapping wing, and right flapping wing, respectively. (B) Flight moment arms. *d*_1_, *d*_2_, *l*_1_, and *l*_2_ are the geometric distances of the 2 rotors and 2 pairs of flapping wings to the nominal CoM. (C and D) The safety attachment model of the robot on the vertical wall. (C) Model of the flipping action around the contact points between the rear belt and the wall. Here, *F*_Ni_, i = {(a), (b), (c)} is the support force of the vertical wall on the contact surface of the robot. *F*_ri_, i = {(1), (2)}, denotes the negative pressure produced by the head and tail rotors. *F*_a_ is the adhesion of the wall to the belt. (D) Integral slipping model. *f*_i_, i = {(1), (2)}, denotes the friction on the belts and battery. *F*_N_ is the total support force of the wall on the robot, and *F*_r_ is the total negative pressure from the rotors. (E) Kinematic model of the robot during climbing. The body frame *x*_b_C*y*_b_ is fixed to the robot with the origin at the nominal CoM. *V*_C_ is the velocity of the robot when climbing upward. (F) Mechanical model of the robot during climbing. *F*_y_ is the frictional force of the robot's belt on the wall. *F*_i_, i = {(L), (R)}, is the driving force of the robot. *F*_P_ is the peeling force of the belt. (G) Process of peeling the adhesive belt from the wall surface. *θ* is the peeling angle, r is the radius of the wheel, and t is the thickness of the belt. The establishment and description of the kinematic and dynamic equations of the robot when flying and climbing are given in the Supplementary Text.

In a gecko, the ventral face of the toes have a dense covering of micro-nano structure setae, each of which generates a weak van der Waals force by contacting the surface using a nano-scale spatula structure. These forces merge into a macroscopic adhesion force, giving the gecko excellent wall adhesion climbing performance [41,42]. At present, biomimetic adhesion materials that are based on the van der Waals force mechanism and that imitate the setae of the gecko’s micro-nano structure can achieve relatively stable adhesion on smooth walls [43]. To develop an aerial–wall robot that can attach to and climb a variety of vertical walls stably, after a comprehensive comparison of various adsorption mechanisms, we adopted a bionic adhesive material in the climbing mechanism. We developed adhesive materials (Text S1, Fig. S2) that can adhere to and detach from various surfaces, and selected a belt-type climbing mechanism that can maximize the adhesive area and improve its adhesion on vertical walls, as shown in Fig. S2D. The rear wheel of the climbing mechanism is the driving wheel, and the whole climbing mechanism weighs 22.5 g. Figure 3C to G presents the kinematics and dynamics models of the aerial–wall robot in the climbing stage.

The design and manufacture of the belt-type climbing module of the aerial–wall amphibious robot give it a low moment of inertia so that it can be attached to the wall and climb flexibly. The adhesive pad attached to the outer surface of the belt provides the adsorption and friction forces when the robot climbs on the wall. If the adhesive performance of the pad is high, it can bear the weight of the whole robot and attach the robot to the wall stably, but the motor cannot produce sufficient torque to drive the belt of the rear wheel to peel it from the wall (Fig. 3G). Eventually, this will increase the climbing load, and then the robot will become trapped on the wall. Therefore, the adhesive pad needs to have both good adhesion and detachment performance. It is used in combination with the negative pressure provided by the rotor to enhance the adhesion. The negative pressure provided by the rotor during wall climbing substantially increases the normal pressure and tangential friction of the contact part and prevents sliding during climbing. Further analysis of the dynamics of climbing is presented in the Supplementary Text.

### Structure design and electronic hardware

To reduce the visibility of the robot, the direction of the rotor motor shaft is designed to face down. The base of the flapping mechanism is fixed to the frame and does not generate vector deflection like the rotor (Fig. 2A). The flapping-wing mechanism is decelerated by 2 stages of the gear set, and the rotating motion is converted into linear motion by a crank linkage mechanism so that the rocker arm drives the flapping wing to reciprocate and generate periodic lift. The geometry and manufacturing process of the wings of the flapping-wing mechanism are described in Ref. [44]. The rigid leading edge and root rods ensure the stability of the flexible wing, enabling the shape and camber of the wing to change passively, similar to the wings of insects during flapping [45,46]. For the complete dynamics and aerodynamics of flapping wings, we refer readers to Ref. [4]. The amplitude of the flapping wing system is determined by the mechanical characteristics of the flapping mechanism, which is fixed at 90°. The flapping frequency is determined by the rotation speed of the motor. The flapping frequency is about 15 Hz when the robot flies in normal speed. The upper limit of the flapping frequency is determined by the strength of the mechanical structure, and we also set a safety limit in the flight control procedures, which is about 20 Hz. The frequency of the flapping wing mechanism on both sides is independently modulated by the flight controller to generate the roll torque (Fig. 2D).

**Fig. 4.**
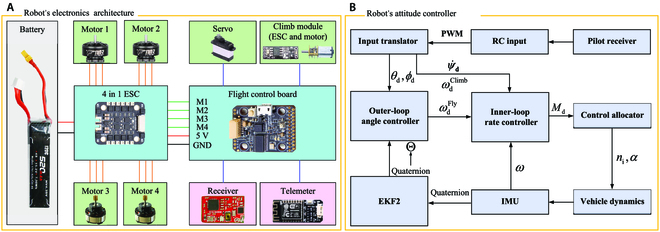
Control architecture of the robot. (A) Main electronics and mechanical components of the robot. (B) Control circuit of the robot. The flying–climbing transition control switches the controllers based on the sensor measurements. *θ*_d_, *φ*_d_, ${\dot{\psi }}_{d}$, $\omega_{d}^{\mathrm{Climb}}$, $\omega_{d}^{\mathrm{Fly}}$, and *M*_d_ are the desired pitch angle, roll angle, yaw angular rate, climbing throttle, flying throttle, and moment to be tracked, respectively. *n*_i_ denotes the input signals to the motors, *α* is the servo deflection angle, *ω* is the attitude angular rate, and Θ contains a sequence of attitude angles.

The overall weight of the aerial–wall robot is 135.38 g. The electronics architecture of the robot is shown in Fig. 4A, and the electronic hardware of the robot is listed in Table S1. The lithium battery is directly connected to a 4-in-1 brushless ESC to power the onboard electronics of the aerial–wall robot. The integrated design of the 4-in-1 brushless ESC greatly simplifies the electronic circuit and reduces the volume and weight occupancy ratio of the electronic equipment. The 4-in-1 ESC supplies power to 4 brushless motors and controls the rotation speed. It also provides a stable 5-V working voltage for the flight controller. The flight controller outputs the control signals processed by flight control operation to each actuator. The control signals of the 4 brushless motors are directly input to the 4-in-1 ESC through a 6-pin line. The robot is also equipped with an AC900 receiver to send the control commands of the remote controller to the flight controller via a single-wire PPM signal.

The flight control board is the core of the whole control system and the key to the continuous and stable flight of the robot. Its main function is to process the signals of various sensors and output the control signals using the computed feedback of the navigation and attitude control system. The stability of the robot attitude is ensured by actuators such as the motor and servo. The flight control board needs to receive signals from the telemeter and receiver as well as output PWM signals (Fig. 4B). A Kakute F7 mini V2 (4 g in weight) was selected as the flight control board of the aerial–wall robot. The onboard STM32F745 processor serves as the main control chip. An onboard MPU6000 inertial measurement unit (IMU) and a BMP280 barometer are used for attitude and altitude measurements, respectively. The details of the control algorithm in the flight controller are described in the Supplementary Text.

### Hybrid power performance of aerial–wall robots

In a flying–climbing amphibious robot, it is very important to have sufficient air and wall control power. Specifically, the hybrid power of the flapping wings and rotor wings of the aerial–wall robot must be able to provide sufficient torque on all 3 axes for attitude control during flight and stability during wall climbing. The control also needs to ensure that the thrust and torque are coupled together to achieve the desired thrust range. In addition, rotor power is important for negative pressure adsorption during climbing. If the constraint of friction force or normal force is not satisfied during the wall climbing, the robot may slide or overturn. To verify that the design parameters of the aerial–wall robot that we selected will provide sufficient control capabilities, we carried out a flapping/rotor hybrid power performance experiment using the robot and evaluated its control capabilities when given control input signals.

**Fig. 5.**
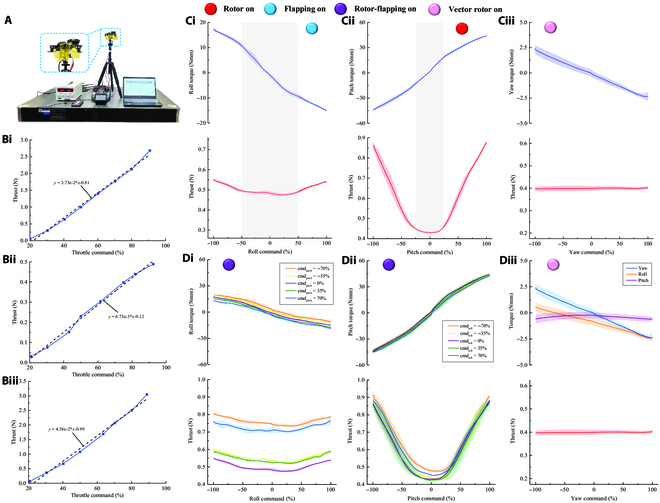
Thrust and attitude torque measurements of the flapping/rotor hybrid power of the aerial–wall robot under various input throttle and roll/pitch/yaw command. (A) Experimental setup for the measurement. The robot was fixed to the sensor. (B) Thrust versus throttle command when only the rotor wings are actuated (i), when only the flapping wings are actuated (ii), and when the combined rotor/flapping wings are actuated (iii). All relationships follow a linear trend (black dashed line) with respective *R*^2^ values of 0.993, 0.993, and 0.989 for measurements respectively carried out with 2 rotors, with the flapping mechanism pair, and with the combined rotor/flapping wings, each driven by the ESC. (C) Torque measurements. (i) Roll torque and the corresponding thrust versus roll command. (ii) Pitch torque and the corresponding thrust versus pitch command. (iii) Yaw torque and the corresponding thrust versus yaw command. The solid lines are the average of 5 measurements, and the shaded area around the mean indicates the standard deviations. The gray shaded areas represent the linear region of the average thrust and torque under some interval commands. (D) Measurements of the roll, pitch, and yaw torques performed at 40% throttle command. (i) Roll torque and the corresponding thrust measurements against the roll command for various values of pitch command, color coded according to the legend. (ii) Pitch torque and the corresponding thrust measurements against the pitch command for various values of roll command, color coded according to the legend. (iii) Yaw, roll, pitch torques, and the corresponding thrust measurements against yaw command, color coded according to the legend. The solid lines are the average of 5 measurements, and the shaded area around the mean indicates the standard deviations. The round pie buttons of different colors present the working state of the flapping wing and rotor.

Figure 5 shows the relationship between the net vertical thrust (or attitude moment) of the aerial–wall robot and the control input signal under the conditions of no wind and no angle of attack. The achievable moments of the robot under different conditions are also shown. Figure 5B shows the thrust values of the aerial–wall robot when executing different power commands under varying throttle input signals. Figure 5B(i) shows that in the rotor power system, the net vertical thrust of the 2 rotors reaches the total weight of the robot at 50% throttle command. Moreover, the thrust force is approximately linear with respect to the throttle command and exceeds 250 g at 90% throttle command. The thrust can increase load carrying capacity and avoid the problem of insufficient lift of the robot. Figure 5B(ii) shows that at 50% throttle command, the net vertical thrust of the 2 pairs of flapping wings driven by 2 motors is approximately 25 g until it reaches half of the total weight of the robot at 90% throttle command. The robot does not need to control the roll over a large angle; in fact, roll should be avoided during wall climbing because it will cause the robot to flip over on the wall, thus interfering with the climbing. Therefore, the thrust generated by the flapping wings is sufficient. Moreover, the thrust generated by the flapping wings and throttle command is approximately linear. Figure 5B(iii) shows that the net vertical thrust of the flapping/rotor hybrid power when actuated maintains an approximately linear relationship with respect to the throttle command, indicating that the brushless motor and the load characteristics of the flapping/rotor hybrid are well matched when the robot is working. Besides, the robot can fly at 60% throttle command. At that moment, its thrust force is 163.8 g, and the thrust–weight ratio is greater than 1. The thrust of flapping wing is 28.4 g, and the proportion of thrust produced by the flapping wing is 17.3%. The power limit is reached at 90% throttle command. At that moment, the overall thrust is 295.2 g, and the thrust of the flapping wing is 48.6 g; thus, its thrust proportion is 16.5%. The power allocation of the rotors and flapping wings is appropriate, and their combination does not weaken the performance of each component. In addition, the flapping wings have higher dynamic efficiency compared with the rotor power: The efficiency of the flapping wing and rotor power is 0.045 N/W and 0.028 N/W, respectively (Fig. S1).

Figure 5C shows the corresponding thrust and torque relative to the independent attitude command in Fig. 5D achieved by the aerial–wall robot. Figure 5C(i) reveals the influence of single roll signal on the thrust and roll moment of the robot without a pitch and yaw control signal. Under a roll command of −50% to 50% (the shaded part in Fig. 5C(i)), the generated roll moment curve has a steeper slope and is linearly related to the roll command. By contrast, the roll moment curve in the non-shaded area is gentle, and there are turning points between the 2 areas. Furthermore, the thrust generated by the robot under the roll command displays a similar phenomenon. A roll command of −50% to 50% does not affect the thrust, but outside of this range, the roll command will produce an additional thrust on the robot. The reason for this phenomenon is that in the range of −50% to 50%, a decrease in the flapping frequency on one side of the horizontal axis corresponds to an increase in the flapping frequency on the other side, which is correspondingly related. However, when the control range is exceeded, the flapping frequency of the flapping wing on one side will be low to idle frequency, while the flapping frequency on the other side continues to increase, which will lead to a gradual decrease in the difference and then a gentle roll moment. Identically, for roll command signals in the range of −50% to 50%, the thrust increases and decreases in the original opposing directions maintain an equal relationship, and hence the difference remains constant. Outside of this range, although the increase in thrust on one side stops, the thrust on the other side continues to decrease, and the net thrust increases. This is related to the values set for the control parameters.

Similarly, under the single pitch moment control signal, the linear correlation between the pitch moment and pitch command is for −25% to 25% pitch command, and the control signal in this region has no effect on the net thrust (Fig. 5C(ii), shaded area). Outside this range, the pitch torque becomes gentle and the thrust increases linearly, mainly because the rotor thrust accounts for the main part of the thrust in the hybrid power. Because the effective moment calculated in this case is with respect to the center of mass (CoM) of the robot rather than the wall contact point, the maximum flying–climbing transition moment is larger because of the shortening of the moment arm.

Figure 5C(iii) shows the yaw moment due to the vector thrust of the rotor. Over the whole range of the individual yaw moment control signal, the yaw moment and command exhibit obvious linear correlation. Even though the tilt angle of the rotor changes, the vertical part still accounts for the majority of the net thrust during flight. We take advantage of this when allocating control power between the 2 rotors. Therefore, the net vertical thrust is also not affected by the yaw control signal, indicating the rationality of controlling the heading.

Figure 5D(i) to (iii) shows the roll, pitch, and yaw moment control signals during the experiment, respectively. Figure 5D(i) and (ii) shows the compound pitch and roll commands executed with 40% throttle command and 0% yaw command. Under each specified pitch command (−70%, −35%, 0%, 35%, and 70%), the roll moment control signal is continuously changed to obtain the torque and thrust under the corresponding command (Fig. 5D(i)). Figure 5C(i) is one of the cases in Fig. 5D(i) at 0% pitch command. The roll torque and thrust obtained by any specified pitch signal follow the same variation rule as in Fig. 5C(i) (linear region and nonlinear region; non-influence region and influence region). Moreover, under the composite control signal, different pitch control signals have no effect on the generated roll torque. Instead, they only influence the generated net thrust, which is the same reason that the rotors dominate the main part of the vertical thrust. From a 3-dimensional (3D) perspective, the thrust curves generated by different pitch control signals under the composite control signals are also parabolas that open upward.

Figure 5D(ii) shows the pitch torque and thrust obtained by continuously changed pitch command at each specified roll command (−70%, −35%, 0%, 35%, and 70%). Figure 5C(ii) is one of the cases in Fig. 5D(ii) at 0% roll command. The pitching torque and thrust obtained by any specified control roll signal follow the same rule shown in Fig. 5C(ii). Moreover, the different roll commands under the composite signal have almost no influence on the pitch thrust generated, just as for the net thrust. In addition to the range of −25% to 25% pitch command, the roll command of ±70% has a thrust increase of less than 0.1 g, which is related to the small contribution of the flapping wings to the thrust in the hybrid power. Only when the roll command is high enough does the net thrust increase slightly.

Figure 5D(iii) shows the relationship between the input of yaw command and the output of the thrust or triaxial thrust under constant pitch and roll commands and 40% throttle command. The change in the yaw command does not affect the net thrust and pitch thrust, and is linearly related to the yaw thrust. The influence of the yaw command on the roll torque of the robot needs to be explained. The roll torque of the robot is also linearly correlated with the yaw command. Although the correlation is smaller than it is for the yaw torque, there are still changes. The rocker arm of the servo drives the rotor to tilt around the longitudinal axis of the fuselage when the designed vector rotor (Fig. 2H) is executing yaw command as the robot’s position is fixed. The arm of the force generated by the rotor and the fixed position is long, which increases the roll torque measured by the sensor. However, during actual flight, the servo shaft is very close to the center of gravity of the fuselage, and hence it does not produce this roll torque in the experiment. This indicates that in addition to yaw torque, the vector servo will produce a low level of pitch and roll torque due to coupling, which can be ignored when compared with the thrust moment. Moreover, the stability compensation of the flight control can guarantee that it does not affect other attitudes, and this was verified in the takeoff and landing experiments in free flight.

The generated roll and pitch torques can thus be decoupled almost completely during actual flight, maintaining an approximately linear function of the command (Fig. 5C and D). Thrust was kept within ±5% of the nominal case value with a zero attitude command in all measurements. The 3-axis torque input and output of the flapping/rotor hybrid power control were proven to be independent and non-interfering. The torque value meets the control demand and the control distribution logic is reasonable, providing a reliable flight platform basis for the evaluation of the flying–climbing transition mechanism.

### Flying–climbing transition performance of aerial–wall robots

**Fig. 6.**
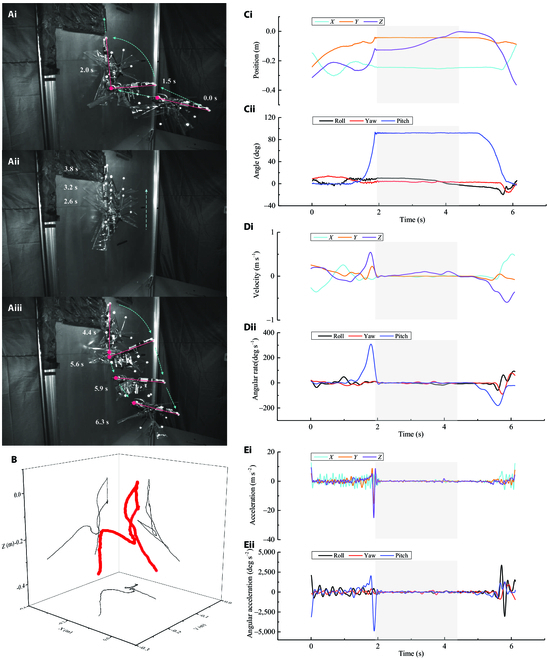
Transitional flight tests of air flight to wall climbing and then to air flight. (A) Composite images of (i) transition from flying to climbing, (ii) climbing, and (iii) transition from climbing to flying. (B) Flight trajectory. (C) Position versus time (i) and body attitude angles versus time (ii). (D) Velocity versus time (i) and body angular rates versus time (ii). (E) Acceleration versus time (i) and body angular accelerations versus time (ii). The solid red lines represent the body of the robot, whose head is indicated by a red dot. The cyan dotted line shows the trend of the robot’s movement. The gray shaded areas represent the corresponding range from completion of the transition from flying to climbing to the end of the climbing phase.

In this experiment, we demonstrated the ability of the aerial–wall robot to transition between flying in the air and climbing on the wall (Fig. 6 and Movie S2). We conducted the experiment in an indoor laboratory, and the whole experiment was manually controlled. The position, velocity, and acceleration of the aerial–wall robot in 1 of the 7 repetitive experiments are shown (Fig. 6C to E). The aerial–wall robot was controlled so that it transitioned from flying to climbing by distributing the thrust of the 2 rotors until the entire robot’s dorsal side was in contact with the wall (*t* = 2.0 s). At this point, an upward velocity began and the robot climbed up the wall. The robot was then controlled to transition from climbing to flying by changing the thrust difference of both rotors again. The results of this experiment demonstrate that the robot can perform controlled maneuvers and smooth transitions between flying, landing, climbing, and takeoff.

Figure 6B illustrates the CoM trajectory of the aerial–wall robot during the whole flying–climbing transition. It moves from the air to the wall and back to the air. The displacement in the *X*, *Y*, and *Z* directions of the trajectory of the robot from finishing the flying–climbing transition to finishing the whole climbing stage (the gray shaded part in Fig. 6) is shown in Fig. 6C(i) (*x*: 0.13 cm, *y*: 0.06 cm, *z*: 12.36 cm). In contrast to the displacement generated in the *Z*-axis, the robot climbs straight up without any lateral deviation on the wall, that is, no deflection in the *X*- or *Y*-axis directions. The attachment–climbing phase takes approximately 2.4 s (gray shaded part, *t* = 2.0 s to *t* = 4.4 s). During the flying–climbing transition, the maximum roll, yaw, and pitch angles of the robot are 19.9°, 16.1°, and 93.4° (Fig. 6C(ii)), respectively, and the robot can be quickly adjusted to a stable climbing position as soon as it lands.

We show that the aerial–wall robot can perform continuous transitions, not just from flying to climbing or from climbing to flying (Fig. 6A and Movie S2). The whole process involves taking off from the ground outside the field of view to hover (50 cm above the ground) and flying forward to a short distance from the wall. Then, the pitch maneuver is triggered, completing the transition from the air to the wall. After that, the robot climbs a short distance (approximately 15 cm), where the climbing speed on the vertical wall is 6 cm/s. Afterwards, it transitions from the wall to the air, and finally rises into the air.

In the approach phase (Fig. 6A(i)), the aerial–wall robot moves toward the wall at a non-uniform speed, and it overcomes its gravity through the thrust generated by the flapping/rotor hybrid power. In the contact phase, vy (perpendicular to the wall direction) decreases to zero (Fig. 6D(i)). The adhesive pad hits the acrylic sheet under the action of the robot’s prepressure to form a seal. The horizontal velocity of the robot immediately drops to zero. Before the contact point has time to slide in the vertical direction, the pitch attitude angle of the robot immediately changes, causing it to tilt counterclockwise around the contact fulcrum of the head, which is controlled by the 2 rotors in the head and tail. When the robot finishes tilting, the effective contact part of the whole belt contacts the wall for the first time, and eventually sticks to the wall in a "headstand" posture with its head down and tail up. On the *Y*-axis, visible oscillations occur when the climbing mechanism hits the wall due to the overall elasticity of the adhesive structure (Fig. 6E(i)). To attach to the wall, the robot needs to provide a horizontal preload to allow the fuselage front end to attach to the wall as a fulcrum. The results indicate that the robot is able to provide sufficient force to preload the adhesive pad (Fig. 6E(i)).

We demonstrate that the robot can perform continuous and complete air–wall–air transitions in 6.1 s. In terms of the transition time, we measured the air–wall process using high-speed images as 0.40 ± 0.03 s (*N* = 7). Moreover, the wall–air transition took 0.70 ± 0.09 s (*N* = 7). The wall–air transition time is nearly twice as long as that of the air–wall transition time because the rotor power is sufficient during the air–wall transition, and the strong negative pressure provides the robot with a large pitch attitude, which can be clearly seen from the pitch angle acceleration of the robot during the air–wall transition in Fig. 6E(ii). By contrast, when the robot performs the wall–air transition, we substantially reduce the power of the tail rotor and appropriately increase the power of the head rotor to avoid excessive energy consumption. As a result, the robot smoothly completes the pitch transition due to the overturning moment around the rear wheel of the climbing mechanism, which is caused by friction with the wall and its own gravity. One important factor for realizing a rapid air–wall transition time is to increase the control distribution ratio of the longitudinal rotor and shorten the rapid change period of the rotor rotating velocity. This enables the robot to have high pitch angle acceleration and generate enough torque to attach the robot to the wall. In addition, the robot is balanced to climb only using the negative pressure of the rotor (the flapping wings are turned off before climbing), which is similar to the way a fly folds its wings and moves only using its legs when climbing on a wall.

To avoid interference between the flapping wing and the wall during the flying–climbing transition as well as the adverse effects caused by its high-frequency vibration during climbing, we automatically activate the flapping-wing off program at a fixed pitch angle, which is based on the measurements of the robot’s attitude by the onboard sensor. The change in pitch angle in Fig. 6C(ii) reflects these results in the process of the air–wall transition. When *t* = 1.8 s, the flapping wing closes and the roll angle of the robot approaches zero. When *t* = 5.6 s, the flapping wing starts and the roll angle changes but immediately returns to its previous value.

We also demonstrated that the aerial–wall robot can land, climb, and take off continuously on a variety of complex vertical surfaces (Fig. 1 and Movie S3). A series of vertical surfaces, including glass, marble, wooden door, elastic cloth, painted iron sheet, and bark, were considered, and the robot was found to be capable of performing a stable flying–climbing transition and climbing on the surfaces after attachment. Table S2 is the statistics of success of the aerial–wall robot flying and climbing on different surfaces. The aerial–wall robot has a higher success rate of transition on the surfaces of acrylic sheet, glass, and marble, followed by a lower success rate on the surfaces of wooden door and painted iron sheet, while the surfaces of soft spray cloth, lime wall, and tree surfaces came with the lowest success rate. It is indicated that the adhesive material is more suitable for the hard smooth wall, while the soft spray cloth is too elastic to contact well. Lime wall and tree surface always contain a lot of dust, which is easily adsorbed on the surface of the adhesive pad, resulting in a decrease in the number of available adhesion. The wall-climbing experiments mainly focused on vertical walls at a certain height, and the surfaces were relatively flat. The robot allows a wider range of wall locomotion by the stabilizing ability of the rotors. Moreover, the aerial–wall robot can still avoid uneven walls by taking off with the current design. The rotor power of the robot results in increased flight speed. We also measured the maximum outdoor flight speed of the robot, which is 6.8 m/s. More information about the performance of the aerial–wall robot has been demonstrated in Table S3.

## Discussion

In this article, we described an aerial–wall flying–climbing amphibious robot that uses insects as the bio-mimetic design objects. The robot is small in size and has good invisibility and high visual charm. It can fly and climb stably on walls with a variety of roughnesses, and can convert smoothly between flying and climbing.

### Advantages with other similar flying–climbing robots

In recent years, other robots similar to aerial–wall robots that can both fly and attach or climb have been proposed in the literature. For example, the robot in Ref. [47], which is based on a quadrotor to generate thrust, can both fly and attach to a wall and it uses wheels to move along the wall. Another robot uses a quadrotor as a carrier, and a clamping mechanism with a new type of dry adhesive is added to the front to enable it to stay on and take off from a smooth vertical wall [48]. On the basis of the quadrotor, the climbing robot in Ref. [40] uses a passive technique to perch on the outdoor surface, climb, and then take off again, thus extending the effective mission duration of the micro-aerial vehicle. It could be the smallest aerial–wall attachment robot proposed to date [49]. It improves the landing accuracy on a magnetic wall surface using an iterative learning control algorithm. The land–air–wall cross-domain robot in [50] uses Gecko behavior as a bionic basis. It can fly in the sky, run on the ground, and attach to different kinds of walls. However, it can only statically adsorb on the wall, and a certain gap between its appearance and that of a gecko remains.

The designs of the previous robots seem to share a concept of aerial–wall motion similar to that of aerial–wall amphibious robots. However, they only use rotors for flight maneuvers without considering the bionic concept. The proposed aerial–wall robot has demonstrated that the flapping/rotor hybrid power system realizes not only flight maneuver but also an air–wall transition directly by distribution of the rotor control. The aerial–wall robot is highly bionic. In addition to its insect-like appearance, it improves the success rate of attachment by decelerating actions before landing. It maintains good stability (roll and yaw angles are less than 3°) during wall–air transitions, even in extreme cases when the pitch angle is close to vertical. In addition, under the synergistic action of adhesive pad and rotor negative pressure, this aerial–wall biomimetic robot can resist large external normal and tangential forces so that the robot can achieve stable adhesion and dynamic climbing on vertical surfaces with various roughness. This extends the working time and expands the monitoring scope, which is impossible for traditional flying robots that use only flapping-wing power or only rotor-wing power [4,32,35,37,40,50,51]. Therefore, we believe that the aerial–wall robot truly combines wall climbing with air flying, extending the movement environment, working space, and mission duration of traditional aircraft.

### Limits and future improvements

Some aspects of the aerial–wall robots still have several limitations. The robots can only climb up vertically along a straight line and lack control of the climbing direction when climbing on the wall. In addition, the balancing ability of the aerial–wall robot during climbing comes at the expense of the continuous operation of the rotors, which results in higher energy consumption than a robot that depends on only a climbing mechanism.

We thus plan to improve the performance of the robot during landing, climbing, and takeoff by studying a series of effective structural and behavioral characteristics of insects. Microscopic hooks and claws will be added in the injection mold of the adhesive pad to truly achieve the synergistic interaction between the hooks and adhesive pads of insects, which will help them adapt to complicated environments. It is necessary to improve the robot's control over the climbing direction, including turning or climbing down along the wall. In addition, we plan to complement the navigation, sensing, autonomous control, and long-distance communication of the aerial–wall prototype in the future, and envision robots that can use machine learning methods to optimize the power allocation during flying–climbing transitions or autonomously detect, identify, and track specific targets.

### Potential applications

The aerial–wall robot has a comprehensive set of functions and a wide range of applications. It will be suitable for multiple types of scenarios such as indoors, outdoors, and narrow alleys. It can fly through a narrow space and arrive indoors for flying or wall-climbing inspection. The scope of application is more extensive than manual work for visual monitoring, environmental detection, and personnel search and rescue. The application of multi-point inspection, repair, or replacement tasks in inaccessible situations is of great significance. Another important application for an aerial–wall robot is making documentaries about outdoor wildlife. The robot can take advantage of its bionic appearance to perch on a tree trunk (Movie S3), approach rare species without disturbing them, and record valuable and difficult-to-obtain scenes. The flying–climbing transition behavior of the aerial–wall robot is very similar to the takeoff and landing behavior of insects [52–54], which can be the inspiration for using the reverse exploration of an insect robot to reveal the potential behavior mechanism of insects.

## Materials and Methods

### Adhesive pad of climbing mechanism

PDMS was selected as the raw material of adhesive pad in the preparation of the adhesive surface layer (Fig. S2). First, it is necessary to clean the aluminum alloy mold. The water is first followed by alcohol to clean the whole surface of the mold. The alcohol is poured out after soaking and washing the mold, and the remaining alcohol is volatilized. The ratio of PDMS liquid to curing agent is 20:1 (total weight is 42 g and the thickness of the finished product is 1.3 mm). The PDMS solution must be stirred in a measuring cup for 3 to 5 min to distribute the curing agent. Then, the measuring cup is placed into a vacuum pump at a negative pressure of 0.8 for 30 min until the bubbles in the solution are removed. It must then be poured on the custom aluminum alloy mold. The upper mold is physically aligned with the lower mold by alignment keys and holes. The platform inside the vacuum drying oven is adjusted to the correct level, and the mold poured with the mixed solution is placed in the vacuum drying oven. The negative pressure is 2.5 for 10 min. Then, the temperature is increased to 80 °C, and the high temperature and low pressure are maintained for 2 h. Finally, it is cooled naturally to room temperature. The upper mold is manually peeled off after the initial cure, and the generated adhesive material is removed from the lower mold. The width, length, and thickness of the generated finished adhesive material are 10 mm, 129 mm, and 1.3 mm, respectively, as shown in Fig. S2D. We apply silicone rubber adhesive to the inner side of the adhesive belt and cover the outer side of the synchronous belt to hold it in place. The adhesive pad moves with the synchronous belt to provide adhesion during wall climbing.

### Thrust and torque experiments of the hybrid power system

We use Nano17 Titanium 6 DOF Force/Torque sensors (ATI Industrial Applications, Inc., Calibration SI-8-0.05) to evaluate the robot’s performance under static and hovering (still air) conditions (force resolution is about 1 mN and torque resolution is about 0.005/0.007 Nmm; Fig. 5A). The whole device was placed on the vibration damping platform (ZPT-F-M), and there was a certain distance between the robot and the platform to ensure that the robot was not affected by the ground effect when it flaps. The sampling frequency of the sensor was set as 1 kHz. More details of thrust and torque measurements can be found in text S13.

### Flying–climbing transition experiments

These experiments were conducted in the Pigeon Flight Training Room of the Nanjing University of Aeronautics and Astronautics, which provides sufficient space for these experiments (L × W × H = 3.7 m × 2.8 m × 2.6 m). Four high-speed cameras (OptiTrack Prime 17W, 1,664 × 1,088 pixels) are installed in the room. The location and attitude of the robot equipped with reflective markers (Figs. S4 and S5) were recorded by constructing a motion capture system. In all experiments, batteries were added to power the robots. The flying–climbing transition test recorded the whole process of landing, climbing, and takeoff on an acrylic sheet (L × W × T = 1.8 m × 0.86 m × 8 mm) at a frame rate of 360 frames per second (fps) and a shutter speed of 2,000 fps. More details can be found in text S14.

### Data processing and analysis

To avoid time differences in data recording, cross-correlation was used to determine the time differences between kinematics recordings and onboard datasets. In addition, all flight experiment results are based on the motion tracking data unless otherwise stated in the analysis of this paper. Each marker was tracked by the image analysis software DLTdv6 (R2019a; MathWorks, Inc.). Tracking quality is monitored by the labeling error parameters of OptiTrack Motive 1.10.3 software (NaturalPoint, Inc.). A single frame with an average labeling error greater than 10 mm is considered an outlier. More details of data processing and analysis can be found in text S16.

## Data Availability

All data needed to evaluate the conclusions in the paper are present in the paper or the Supplementary Materials.

## Supplementary Materials

Supplementary 1 Supplementary Text Sections S1 to S16 Figs. S1 to S6 Tables S1 to S3 Movies S1 to S4 References [55–64]

## References

[1] Mountcastle AM, Ravi S, Combes SA. Nectar vs. pollen loading affects the tradeoff between flight stability and maneuverability in bumblebees. Proc Natl Acad Sci USA. 2015;112:10527–10532. 26240364 10.1073/pnas.1506126112 PMC4547240

[2] Crall JD, Chang JJ, Oppenheimer RL, Combes SA. Foraging in an unsteady world: Bumblebee flight performance in field-realistic turbulence. Interface Focus. 2017;7: Article 20160086. 28163878 10.1098/rsfs.2016.0086 PMC5206605

[3] Ravi S, Kolomenskiy D, Engels T, Schneider K, Wang C, Sesterhenn J, Liu H. Bumblebees minimize control challenges by combining active and passive modes in unsteady winds. Sci Rep. 2016;6:35043. 27752047 10.1038/srep35043 PMC5067513

[4] Karásek M, Muijres FT, Wagter CD, Remes BDW, Croon GCHE. A tailless aerial robotic flapper reveals that flies use torque coupling in rapid banked turns. Science. 2018;361(6407):1089–1094. 30213907 10.1126/science.aat0350

[5] Woodward MA, Sitti M. Morphological intelligence counters foot slipping in the desert locust and dynamic robots. Proc Natl Acad Sci USA. 2018;115:E8358–E8367. 30135101 10.1073/pnas.1804239115 PMC6130395

[6] Ji A, Han L, Dai Z. Adhesive contact in animal: Morphology, mechanism and bio-inspired application. J Bionic Eng. 2011;8:345–356.

[7] Dai Z, Gorb SN, Schwarz U. Roughness-dependent friction force of the tarsal claw system in the beetle *Pachnoda marginata* (Coleoptera, Scarabaeidae). J Exp Biol. 2002;205:2479–2488. 12124371 10.1242/jeb.205.16.2479

[8] Meng X, Liu Y, Sun M. Aerodynamics of ascending flight in fruit flies. J Bionic Eng. 2017;14:75–87.

[9] Liang B, Sun M. Aerodynamic interactions between wing and body of a model insect in forward flight and maneuvers. J Bionic Eng. 2013;10(1):19–27.

[10] Wang X, Wu Z. Stroke-averaged lift forces due to vortex rings and their mutual interactions for a flapping flight model. J Fluid Mech. 2010;654:453–472.

[11] Ristroph L, Bergou AJ, Ristroph G, Coumes K, Berman GJ, Guckenheimer J, Wang ZJ, Cohen I. Discovering the flight autostabilizer of fruit flies by inducing aerial stumbles. Proc Natl Acad Sci USA. 2010;107:4820–4824. 20194789 10.1073/pnas.1000615107 PMC2841947

[12] Muijres FT, Elzinga MJ, Melis JM, Dickinson MH. Flies evade looming targets by executing rapid visually directed banked turns. Science. 2014;344:172–177. 24723606 10.1126/science.1248955

[13] Federle W, Riehle M, Curtis ASG, Full RJ. An integrative study of insect adhesion: Mechanics and wet adhesion of pretarsal pads in ants. Integr Comp Biol. 2002;42:1100–1106. 21680393 10.1093/icb/42.6.1100

[14] Song Y, Dai Z, Wang Z, Ji A, Gorb SN. The synergy between the insect-inspired claws and adhesive pads increases the attachment ability on various rough surfaces. Sci Rep. 2016;6:26219–26227. 27198650 10.1038/srep26219 PMC4873747

[15] Asbeck AT, Kim S, Cutkosky MR, Provancher WR, Lanzetta M. Scaling hard vertical surfaces with compliant microspine arrays. Int J Robot Res. 2006;25:1165–1179.

[16] Xu F, Wang X, Jiang G. Design and analysis of a wall-climbing robot based on a mechanism utilizing hook-like claws. Int J Adv Robot Syst. 2012;9(261):1–12.

[17] Ji A, Zhao Z, Manoonpong P, Wang W, Chen G, Dai Z. A bio-inspired climbing robot with flexible pads and claws. J Bionic Eng. 2018;15:368–378.

[18] Daltorio KA, Wei TE, Horchler AD, Southard L, Wile GD, Quinn RD, Gorb SN, Ritzmann RE. Mini-whegs TM climbs steep surfaces using insect-inspired attachment mechanisms. Int J Robot Res. 2009;28:285–302.

[19] Kim S, Spenko M, Trujillo S, Heyneman B, Santos D, Cutkosky MR. Smooth vertical surface climbing with directional adhesion. IEEE Trans Robot. 2008;24(1):65–74.

[20] Henrey M, Ahmed A, Boscariol P, Shannon L, Menon C. Abigaille-III: A versatile, bioinspired hexapod for scaling smooth vertical surfaces. J Bionic Eng. 2014;11:1–17.

[21] Xu Z, Ma P. A wall-climbing robot for labelling scale of oil tank’s volume. Robotica. 2002;20(2):209–212.

[22] Lal TR, Mukherjee R, Xi N, Aslam D, Dulimarta H, Xiao J, Minor M, Dang G. Climbing the walls [robots]. IEEE Robot Autom Mag. 2002;9(4):10–19.

[23] Zhu J, Sun D, Tso SK. Development of a tracked climbing robot. J Intell Robot Syst. 2002;35:427–443.

[24] Zufferey J-C, Klaptocz A, Beyeler A, Nicoud J-D, Floreano D. A 10-gram vision-based flying robot. Adv Robot. 2007;21:1671–1684.

[25] Kim HJ, Kim M, Lim H, Park C, Yoon S, Lee D, Choi H, Oh G, Park J, Kim Y. Fully autonomous vision-based net-recovery landing system for a fixed-wing UAV. IEEE ASME Trans Mech. 2013;18(4):1320–1333.

[26] Crowther B, Lanzon A, Maya-Gonzalez M, Langkamp D. Kinematic analysis and control design for a nonplanar multirotor vehicle. J Guid Control Dyn. 2011;34:1157–1171.

[27] Mahony R, Kumar V, Corke P. Multirotor aerial vehicles: Modeling, estimation, and control of quadrotor. IEEE Robot Autom Mag. 2012;19:20–32.

[28] Brescianini D, D’Andrea R. An omni-directional multirotor vehicle. Mechatronics. 2018;55:76–93.

[29] Ma KY, Chirarattananon P, Fuller SB, Wood RJ. Controlled flight of a biologically inspired, insect-scale robot. Science. 2013;340:603–607. 23641114 10.1126/science.1231806

[30] Phan HV, Au TKL, Park HC. Clap-and-fling mechanism in a hovering insect-like two-winged flapping-wing micro air vehicle. R Soc Open Sci. 2016;3: Article 160746. 28083112 10.1098/rsos.160746 PMC5210694

[31] Jafferis NT, Helbling EF, Karpelson M, Wood RJ. Untethered flight of an insect-sized flapping-wing microscale aerial vehicle. Nature. 2019;570:491–495. 31243384 10.1038/s41586-019-1322-0

[32] Phan HV, Park HC. Mechanisms of collision recovery in flying beetles and flapping-wing robots. Science. 2020;370:1214–1219. 33273101 10.1126/science.abd3285

[33] Dudek G, Giguere P, Prahacs C, Saunderson S, Sattar J, Torres-Mendez L-A, Jenkin M, German A, Hogue A, Ripsman A, et al. Aqua: An amphibious autonomous robot. Computer. 2007;40:46–53.

[34] Ijspeert AJ, Crespi A, Ryczko D, Cabelguen J-M. From swimming to walking with a salamander robot driven by a spinal cord model. Science. 2007;315:1416–1420. 17347441 10.1126/science.1138353

[35] Li L, Wang S, Zhang Y, Song S, Wang C, Tan S, Zhao W, Wang G, Sun W, Yang F, et al. Aerial-aquatic robots capable of crossing the air-water boundary and hitchhiking on surfaces. Sci Robot. 2022;7(66): Article eabm6695. 35584203 10.1126/scirobotics.abm6695

[36] Peterson K, Birkmeyer P, Dudley R, Fearing R. A wing-assisted running robot and implications for avian flight evolution. Bioinspir Biomim. 2011;6(4): Article 046008. 22004831 10.1088/1748-3182/6/4/046008

[37] Kim K, Spieler P, Lupu E-S, Ramezani A, Chung S-J. A bipedal walking robot that can fly, slackline, and skateboard. Sci Robot. 2021;6(59): Article eabf8136. 34613821 10.1126/scirobotics.abf8136

[38] Desbiens AL, Asbeck AT, Cutkosky MR. Landing, perching and taking off from vertical surfaces. Int J Robot Res. 2011;30(3):355–370.

[39] Dickson JD, Clark JE. Design of a multimodal climbing and gliding robotic platform. IEEE ASME Trans Mech. 2013;18(2):494–505.

[40] Pope MT, Kimes CW, Jiang H, Hawkes EW, Estrada MA, Kerst CF, Roderick WR, Han AK, Christensen DL, Cutkosky MR. A multimodal robot for perching and climbing on vertical outdoor surfaces. IEEE Trans Robot. 2017;33(1):38–48.

[41] Autumn K, Sitti M, Liang YA, Peattie AM, Hansen WR, Sponberg S, Kenny TW, Fearing R, Israelachvili JN, Full RJ. Evidence for van der Waals adhesion in gecko setae. Proc Natl Acad Sci USA. 2002;99(19):12252–12256. 12198184 10.1073/pnas.192252799 PMC129431

[42] Luo Y, Chen X, Tian H, Li X, Lu Y, Liu Y, Shao J. Gecko-inspired slant hierarchical microstructure-based ultrasensitive iontronic pressure sensor for intelligent interaction. Research. 2022;2022: Article 9852138. 35935142 10.34133/2022/9852138 PMC9275085

[43] Kamperman M, Kroner E, Campo A, McMeeking RM, Arzt E. Functional adhesive surfaces with “gecko” effect: The concept of contact splitting. Adv Eng Mater. 2010;12(5):335–348.

[44] Li Q, Ji A, Shen H, Li R, Liu K, Zheng X, Shen L, Han Q. Experimental study on the wing parameter optimization of flapping-wing aircraft based on the clap-and-fling mechanism. Int J Aeronaut Space Sci. 2022;23:265–276.

[45] Bergou AJ, Xu S, Wang Z. Passive wing pitch reversal in insect flight. J Fluid Mech. 2007;591:321–337.

[46] Dickinson MH. Insect flight. Curr Biol. 2006;16(9):R309–R314. 16682333 10.1016/j.cub.2006.03.087

[47] Shin J-U, Kim D, Kim J-H, Myung H, Micro aerial vehicle type wall-climbing robot mechanism. Paper presented at: 22nd IEEE International Symposium on Robot and Human Interactive Communication; 2013 Aug 26–29; Gyeongju, Korea.

[48] Kalantari A, Mahajan K, Ruffatto D, Spenko M, Autonomous perching and take-off on vertical walls for a quadrotor micro air vehicle. Paper presented at: 2015 IEEE International Conference on Robotics and Automation; 2015 May 26–30; Seattle, WA.

[49] Chirarattananon P, Ma KY, Wood RJ. Perching with a robotic insect using adaptive tracking control and iterative learning control. Int J Robot Res. 2016;35(10):1185–1206.

[50] Huang C, Liu Y, Wang K, Bai B. Land–air–wall cross-domain robot based on gecko landing bionic behavior: System design, modeling, and experiment. Appl Sci. 2022;12(8):3988.

[51] Desbiens AL, Cutkosky MR. Landing and perching on vertical surfaces with microspines for small unmanned air vehicles. J Intell Robot Syst. 2010;57(1–4):313–327.

[52] Balebail S, Raja SK, Sane SP. Landing maneuvers of houseflies on vertical and inverted surfaces. PLOS ONE. 2019;14: Article e0219861. 31412069 10.1371/journal.pone.0219861 PMC6693754

[53] Liu P, Sane SP, Mongeau J-M, Zhao J, Cheng B. Flies land upside down on a ceiling using rapid visually mediated rotational maneuvers. Sci Adv. 2019;5:eaax1877. 31681844 10.1126/sciadv.aax1877 PMC6810462

[54] Card G, Dickinson MH. Performance trade-offs in the flight initiation of drosophila. J Exp Biol. 2008;211(Pt 3):341–353. 18203989 10.1242/jeb.012682

[55] Dickinson MH, Lehmann F-O, Sane SP. Wing rotation and the aerodynamic basis of insect flight. Science. 1999;284:1954–1960. 10373107 10.1126/science.284.5422.1954

[56] Liu J, Xu L, Xu J, Li T, Chen S, Xu H, Cheng G, Ceccarelli M. Design, modeling and experimentation of a biomimetic wall-climbing robot for multiple surfaces. J Bionic Eng. 2020;17:523–538.

[57] Kendall K. Thin-film peeling-the elastic term. J Phys D Appl Phys. 1975;8:1449–1452.

[58] Lee CH, Kim DR, Cho IS, William N, Wang Q, Zheng X. Peel-and-stick: Fabricating thin film solar cell on universal substrates. Sci Rep. 2012;2:1000. 23277871 10.1038/srep01000 PMC3533453

[59] Kim J, Kim KS, Kim YH. Mechanical effects in peel adhesion test. J Adhes Sci Technol. 1989;3:175–187.

[60] Kim J, Kim DW, Baik S, Hwang GW, Kim T, Pang C. Snail-inspired dry adhesive with embedded microstructures for enhancement of energy dissipation. Adv Mater Technol. 2019;4:1900316.

[61] Chin Y-W, Kok JM, Zhu Y-Q, Chan W-L, Chahl JS, Khoo BC, Lau G-K. Efficient flapping wing drone arrests high-speed flight using post-stall soaring. Sci Robot. 2020;5(44):eaba2386. 33022610 10.1126/scirobotics.aba2386

[62] Hedrick TL. Software techniques for two- and three-dimensional kinematic measurements of biological and biomimetic systems. Bioinspir Biomim. 2008;3: Article 034001. 18591738 10.1088/1748-3182/3/3/034001

[63] Stevens BL, Lewis FL. *Aircraft control and simulation*. New York (NY): Wiley-Interscience Press; 2003.

[64] Craig JJ. *Introduction to robotics: Mechanics and control*. Boston (MA): Pearson Education Inc.; 1986.

