# Supplementary material for: An Aerial–Wall Robotic Insect That Can Land, Climb, and Take Off from Vertical Surfaces
Source: Research (Wash D C). 2023 May 10;6:0144. doi: 10.34133/research.0144 (PMC10204747; doi:10.34133/research.0144)
Supplement: Supplementary 1 — Supplementary Text Sections S1 to S16 Figs. S1 to S6 Tables S1 to S3 Movies S1 to S4 References [55–64] [file research.0144.f1.zip › Revised Supplemental Material (Clean Version).docx]

Supplementary Materials

Supplementary Text

Sections S1 to S16

Figs. S1 to S6

Tables S1 to S3

Movies S1 to S4

**Supplementary Text**

**Section S1. Definition of the coordinate system**

The coordinate system should be established before the dynamic model. The ground coordinate system is fixed to the earth and is denoted as , where the -axis points east, the -axis points vertically up, and the -axis points north according to the right-hand rule. The body coordinate system is fixed to the fuselage and is denoted as . The origin of the body coordinate system coincides with the center of gravity of the fuselage, the -axis points to the right side of the fuselage, the -axis is perpendicular to the plane of the fuselage, and the -axis is oriented toward the head direction according to the right-hand rule.

**Section S2.** **Newton–Euler equation**

The robot is regarded as a rigid body for dynamic modeling. According to the Newton–Euler equation, the motion of a rigid body can be decomposed into translation of the center of mass (CoM) and rotation around the CoM. The general form is

(1)

where is the resultant of all external forces on the robot, is the resultant of all external torques on the robot, is the velocity of the robot, is the angular velocity of the robot, and is the inertia matrix of the robot. To analyze the position and attitude of the robot, the force and translation are expressed in the ground coordinate system , and the moment and rotation are expressed in the body coordinate system . Then, the Newton–Euler equation can be transformed into

(2)

where is the total thrust of the rotors and flapping wings; is the total torque of the rotors and flapping wings; represents the gravity in the ground coordinate system ; and respectively represent the uncertain force and moment caused by airflow interference, reverse torque, irregular vibration, and other factors; and is the moment of inertia of the robot. Finally, represents the rotation matrix of the robot body coordinate system converted to the ground coordinate system.

**Section S3. Actuator forces and torques**

Unlike fixed-wing, rotary-wing, or flapping-wing aircraft, which use traditional actuators, the aerial–wall robot simultaneously generates control force and torque through hybrid power consisting of devices such as rotors, flapping wings, and servos to manipulate the robot. The aerial–wall robotic insect has six actuators, which are arranged symmetrically in the shape of a cross, and consist of the left and right flapping-wing power group, head and tail rotor power group, vector deflection servo, and climbing power group. The two sets of rotors rotate in opposite directions, and hence the reverse torque caused by rotor resistance is reversed. Reverse torque has little effect on flight maneuvering and is therefore classified as an indeterminate torque during the following modeling. In addition, the aerodynamic characteristics of the flapping wing are more complex and nonlinear than those of conventional actuators (*55*). However, the actual tests revealed that the lift provided by the flapping-wing power group is proportional to the flapping frequency.

The simple mechanical models of the robot during flight are presented in Fig. 3A and 3B. The thrust generated by the head and tail rotors are expressed as and , respectively. The thrust generated by the left and right flapping wings are expressed as and , respectively, and the deflection angle that drives the servo is expressed as . The distance from the head rotor to the CoM of the robot is *d*1, and the distance from the tail rotor to the CoM of the robot is *d*2. The thrust point of action of the two sets of flapping wings is half of the centerline of the leading edge of the flapping wing from the wing root, and the distances between the action points and the CoM are *l*1 and *l*2.

Hence, the total thrust of the robot is

(3)

In addition, the total torque received by the robot is

(4)

**Section S4. Attitude kinematics of the robot**

The projection of the angular velocity of the robot on the body coordinate system is

|  | (5) |
| --- | --- |

The attitude of the robot in flight is generally determined by three Euler angles, which are constantly changing during flight and are related to the rotation angular rate of the robot in all directions. The kinematic equation of the robot’s rotation around the CoM can be obtained by establishing the relationships between them. According to the projection of the rotation angular rate on the robot, the following equations can be obtained as

|  | (6) |
| --- | --- |

Solving these equations obtains the following kinematic equations of the robot rotating around the CoM as

|  | (7) |
| --- | --- |

**Section S5. Climbing statics**

The negative pressure generated by the rotor causes the aerial–wall robot to pitch substantially and attach to the vertical wall. As the suction of the negative pressure adsorption system increases, the stability of the robot’s movement on the wall increases, but its kinematic dexterity will decrease and the power consumption will increase accordingly. To quantitatively reveal the safe attachment conditions of the robot on the wall, it is necessary to analyze the form of the potential instability during this movement to determine the relationship between the adsorption force of the negative pressure system and the gravity, wall friction coefficient, and other parameters.

Statics is used to analyze the stability of the aerial–wall robot when it attaches to the vertical wall after completing the flying–climbing transition. Because the transition wall is vertical, the main instability form of the robot is overturning around the contact line between the rear belt and the wall (*56*). The whole robot will slip along the wall if the negative pressure provided by the rotor of the robot head is small.

As shown in Fig. 3C, the main reason for overturning around the rear belt is that the distance between the center of gravity and the wall is the largest in the vertical wall direction, and hence the robot has the greatest probability of overturning backward. To ensure the safe and stable operation of the robot, the support force of the tail battery of the robot and the upper belt must meet the conditions and . From the force and moment balance equation of the robot, it can be seen that

|  | (8) |
| --- | --- |

where *G* is the gravity acting on the aerial–wall robot, is the support force of the wall on the tail battery, is the support force of the wall on the upper side of the adhesive belt, is the support force of the wall on the lower side of the adhesive belt, is the adsorption force generated by the tail rotor, is the adsorption force generated by the head rotor, is the adhesion force of the wall to the adhesive belt, is the distance from the center of gravity to the wall, is the distance from the center of the tail rotor to the center of the rear belt wheel, is the distance between the center of the front and rear belt wheels, and is the distance from the center of the head rotor to the center of the rear belt wheel.

From Equation (8), the following conditions can be derived to determine the adsorption force that should be generated by the head and tail rotors

|  | (9) |
| --- | --- |

As shown in Fig. 3D, the whole robot will slip if the friction between the wall and the belts on both sides as well as the tail batteries cannot overcome the component force of the robot’s gravity along the wall. In this figure, is the total friction force on the left and right belts and is the friction force on the tail battery in the opposite direction to the component force of gravity on the wall. Moreover, is the total support force of the wall on the robot, assuming that the friction coefficient between the belt and the wall is , the friction coefficient between the tail battery and the wall is . Finally, is the total negative pressure generated by the rotor. To prevent the robot from sliding along the wall, the friction force should meet the following conditions

|  | (10) |
| --- | --- |

The friction force can also be approximately expressed as , where is the equivalent friction coefficient. As , it can be obtained that the adsorption force of the negative pressure adsorption system should meet the following condition

|  | (11) |
| --- | --- |

**Section S6. Climbing kinematics and dynamics**

When the aerial–wall robot contacts the wall, forward friction will be generated. However, in addition to friction, the robot on the wall will be affected by the adhesive force of the belt. To further study the motion track of the robot during wall climbing and the relationship of the change in the system mechanics, the kinematics and dynamics of the climbing movement of the robot should be deeply analyzed to optimize the structure of the robot and implement a control strategy. When the robot climbs vertically, the two rear wheels provide driving force, and the front wheels are not subject to friction. The flapping wings are closed during climbing.

This robot only climbs in the original direction and does not consider turning, lateral sliding, or turning around the left and right belts when climbing. Therefore, only its movement along the vertical axis needs to be considered when analyzing its kinematics during wall climbing. The motion model of the robot during wall climbing is shown in Fig. 3E.

In Fig. 3E, represents the rotation center of the left belt, represents the rotation center of the right belt, b represents the center distance between the left and right belts of the robot, and L represents the effective contact length between the belt and the wall. The kinematics equations of the robot in the body coordinate system *x*b*o*b*y*b are

|  | (12) |
| --- | --- |

During wall climbing, the control quantity should be the revolving speed of the driving belt wheel on both sides. According to the triangle geometric relationship in Fig. 3

|  | (13) |
| --- | --- |

where is the rotational angular velocity of the left adhesive belt wheel, is the rotational angular velocity of the right adhesive belt wheel, and r is the radius of the belt wheel.

If Equation (13) is substituted into Equation (12), the kinematics equation of the robot can be converted into

|  | (14) |
| --- | --- |

That is, the robot only has the velocity in the *y* direction about .

The force model of the robot is shown in Fig. 3F. The friction force of the wall surface on the adhesive belt must first be calculated to establish the dynamic model for the climbing mechanism. All stress points are assumed to be applied on the belt to simplify the calculation.

Taking a single belt as an example for force analysis, the friction force of the belt in unit area is

|  | (15) |
| --- | --- |

where B is the belt width and is the wall friction force on the belt in the area of . Then, the friction force in the *y* direction is

|  | (16) |
| --- | --- |

To simplify the calculation of the friction force, assuming that the forces on both sides of the belts are uniform, the force in the *y* direction of the friction force is simplified as

|  | (17) |
| --- | --- |

In addition to the friction force between the wall and the belt, the force of the robot on the wall is affected by its gravity and the driving forces and of the left and right belts. According to the force model of the robot in Fig. 3F, the Newton–Euler equation can be used to obtain the following dynamic equations of the robot in the coordinate system *x*b*o*b*y*b as

|  | (18) |
| --- | --- |

where m is the overall mass of the robot, G is the gravity, and is the peeling force of the belt.

The climbing movement involves the adhesion of the adhesive pad at the front wheel and its detachment at the rear wheel (the peeling of the adhesive material from the wall). Using the physical phenomenon of peeling elastomer off a wall, Kendall and other researchers have analyzed the relationship between the peeling force, elastic modulus E, and peeling angle based on the transformation of the energy of the elastomer in the peeling process (*57–59*). The peeling process of the belt from the wall is shown in Fig. 3G, where is the peeling force, is the peeling angle, r is the radius of the belt wheel, and t is the thickness of the belt.

According to the trigonometric geometric relationship shown in Fig. 3G, the original length of belt section AB and length after stretching are respectively as

|  | (19) |
| --- | --- |

According to the linear elastic theory of belts

|  | (20) |
| --- | --- |

where E is the elastic modulus of the belt, B is the width of belt, and is the increment in belt section length.

The peeling force can be derived from Equations (19) and (20) as

|  | (21) |
| --- | --- |

From Kendall’s adhesive belt theory, we can deduce that during the peeling process of the belt from point B to point C, the change in energy is as follows: 1) The surface energy generated by the new surface peeling is , wherein is the surface energy at the junction between the adhesive belt and wall peeling. 2) The potential energy term caused by the movement of the applied force is . 3) The elastic potential energy caused by the elastic deformation of the adhesive belt in the direction of the applied force is .

The following equation can be obtained by adding the energy of the adhesive belt during peeling

|  | (22) |
| --- | --- |

Therefore, the driving torque T of the motor when the robot moves at a uniform speed is

|  | (23) |
| --- | --- |

where the distance between the center of gravity of the robot and the wall is , and the corresponding peeling force is .

**Section S7. Performance of adhesive materials**

The experiments of adhesion and detachment strength we carried out were referred to the method described in (*57, 59, 60*). All the forces were measured using tension gauge (HANDPI, Yueqing Handpi Instruments Co.,Ltd., China). Normal and tangential pull-off adhesion tests were performed with samples at 2 × 2 cm2 attached to a PVC wafer substrate, which was connected to the tension gauge by a custom fixture. The adhesive side of the adhesive sample was firstly put on the wall surface under 0.75 N/cm2 of preload for 5 s. Then the tension gauge was moved at the speed of 0.1 mm/s. Peel-off adhesion tests were conducted on different surfaces with samples at 1 × 4 cm2, which was carried out with a specially designed peel fixture to maintain a 90° peeling direction during peeling of the entire sample. The tension gauge was moved at 1 mm/s peeling velocity. The pull-off and peel-off force was recorded as the absolute value of the maximum force during a one-time pull. We repeated all measurements at least 10 times at ambient temperature of 25℃, normal pressure at 105 Pa, and relative humidity of about 50%. For each data point, at least three strips of samples were tested and the measured adhesion strength and detachment strength values were averaged. The experimental results of adhesion performance on different surfaces were shown in Fig. S3.

In our measurement, the adhesion strengths of the adhesive material on glass, acrylic sheet, and wooden door surface is highest in pulling and peeling directions (Fig. S3). As contact surface is altered, the measured adhesion strengths in normal and tangential pulling or peeling direction improved with decreasing surface roughness, which is also a factor in the success of the flying-climbing transition (Table S2).

**Section S8. Controller structure**

The control algorithm of the aerial–wall robot is based on the Ardupilot firmware, whose controller configuration is similar to that of the traditional multi-rotor aircraft, as shown in Fig. 4B. The pilot gives flight manipulation commands according to the state of the robot and task requirements. The input signal converter converts the receiver’s signal into the desired pitch angle, desired roll angle, desired yaw angular rate, and desired throttle. The inertial measurement unit (IMU) on the flight control board can measure the attitude data of the robot, in which the estimated angular velocity is directly sent to the attitude controller after processing. In addition, the estimated attitude angle is sent to an extended Kalman filter (EKF) in the form of a quaternion for data fusion and then input into the angle loop in the form of Euler angle. To make the data measurement of IMU sensor closer to the real value, the quaternion-based EKF algorithm is used to estimate attitude. The algorithm uses the EKF to fuse the data of each sensor and estimate the attitude quaternion and gyroscope deviation. The quaternion representation of the attitude can avoid the problem of gimbal lock when the Euler angle is used and the problem of excessive computation when the rotation matrix is used.

**Section S9.** **Attitude controller**

The attitude controller uses the traditional cascade PID control law to maintain the stability of the flight. The outer loop of the feedback loop is the angle control loop, and the inner loop is the angular rate control loop. For the outer loop, we retain the proportional term P and omit the integral term I and the differential term D. On the one hand, the noise introduced by the differential term D can be avoided to reduce its adverse effect on the system, and on the other hand, the proportional term P of the outer loop is sufficient for the system to use and easier to adjust when the inner loop is dominant. In addition, the inner loop is used to suppress the disturbance and improve the response speed of the whole system. In particular, the control law of the outer loop is

(24)

where is the desired angular rate to be fed into the inner loop, is the proportional coefficient, and the attitude angle error is , where is the desired attitude angle, and is the estimated attitude angle. The outer loop inputs the calculated desired angular rate into the inner loop .

For the inner loop, the aerial–wall robot uses angular velocity as the controlled target. In particular, the control law of the inner loop is

(25)

where is the estimated angular rate, is the proportional coefficient, is the integral coefficient, is the differential coefficient, and , where is the desired angular rate. The inner loop inputs the desired torque to the control allocator.

We adjusted the attitude control parameters and improved the flight stability through numerical simulation and flight test verification. Using the approach of first setting the inner loop control parameters and then the outer loop control parameters, the control parameters of each channel were determined by applying a unit step signal to observe the response performance of each channel.

**Section S10. Control allocator**

Next, we derive the control allocation logic of the aerial–wall robot by analyzing the relationship between the actuator movement and the robot dynamics. In the thrust moment of the actuator, the yaw moment is determined by the thrust of the head rotor and the deflection angle of the servo , but is dominant. Because of the particularity of yaw control, yaw is put under individual control. The expected servo deflection angle can be expressed as

(26)

where represents the yaw control allocation coefficient and represents the expected yaw moment. To control of the remaining three degrees of freedom, the four actuator thrust forces are extracted, and the relationship can be written in the form of matrix equation as

(27)

The constant values , , , and are substituted into Equation (27), and the values in each row are normalized using proportional scaling. Then, the coefficient matrix in the formula can be written as

(28)

The Moore–Penrose generalized inverse matrix with parameter is calculated for the coefficient matrix as

(29)

Then, the control allocation equation of the actuator is

(30)

The generalized inverse matrix takes into account the deflection angle of the servo , removes the coupling influence of the head rotor deflection on the lift and pitch moment, and provides a more stable and accurate control allocation logic for the robot.

**Section S11.** **Control** **allocation logic**

When the servo is in a neutral position, , and hence the generalized inverse matrix is

(31)

The specific values of can be understood from the basic control logic of the robot (Fig. 2C–2E). The signal of the throttle channel is synchronously assigned to the left and right flapping-wing power as well as the head and tail rotor power, which together generate lift force and control the lifting movement of the robot. The pitch torque is generated by the differential rotation of the head and tail rotor power, which controls the pitch motion and longitudinal motion of the robot. The differential flapping of the left and right flapping wings generates roll torque, which controls the roll and lateral motions of the robot. In addition, the vector deflection servo drives the deflection of the head rotor power, which generates the yaw torque and controls the yaw motion of the robot.

Note that according to the needs of different scenarios, we can flexibly adjust the power ratio of the rotors and flapping wings, so as to adjust the power distribution of rotors and flapping wings during flight, as well as the overall vibration level of the robot.

**Section S12. Flying-climbing transition strategy**

The desired torque of the Acro mode is mapped to the control allocation output during the flying–climbing transition. The control allocation equation in the latter period of the flying–climbing transition is the same as that of the climbing stage

(32)

Note that if the critical angle is too small, the roll motion of the robot will be out of control for a longer time after the flapping wings are closed. If the critical angle is too large, the flapping wings will flap for a longer time, which will affect the stability of the robot when it is in contact with the wall. The specific value of the critical angle is determined according to this principle.

The RC for the robot sets the angular velocity value when the robot is in the Acro mode and the robot performs angular velocity control. For example, when the RC stick is in the middle, the value of the angular velocity is set to zero instead of setting the value of the roll angle to zero. In other words, when controlling of angular velocity, the change in angle of the robot is not important. In the Stabilize mode, the RC sends the robot the angle information, that is, the angle setting value, and the robot controls its angle. For example, when the RC stick is in the middle, the target roll angle is zero, and hence the robot will be naturally horizontal.

**Section S13.** **Thrust and torque experiments of the hybrid power system of the robot**

We used an external power supply (E36103A, Keysight) to excite the robot system to ensure constant conditions during measurements. The remote sensing function was used to compensate the voltage drop of the power supply cable, and the voltage of the robot was kept near the preset value of 8 V.

The hovering (still air) test consists of two parts. The first is the lift test for hybrid design, which tests the corresponding relationship between throttle input and thrust under the conditions of pure rotor power, pure flapping-wing power, and flapping/rotor hybrid power, respectively. The second is the torque test for hybrid design. Under the constant 40% throttle and yaw command, the relationship between pitch (or roll) input and three-axis torque output was tested. The relationship between yaw input and triaxial torque output was tested under constant 40% throttle and pitch (or roll) command. The definition of the efficiency of the flapping/rotor hybrid power here is the ratio of flapping/rotor thrust to electric power in reference to the expression in (*61*), which could be expressed as η = T/IU, where T is the thrust of the robot and IU is the input electric power.

The collected data were postprocessed using MATLAB 2019a software (MathWorks, Inc.). For the static measurement (constant command, Fig. 5), we report the average of the 2-s measurement. Force and torque signals were low-pass filtered with a fourth-order zero-phase Butterworth filter with a 5 Hz cutoff frequency. This filters out most of the variation due to flapping and vibration caused by flapping while keeping the low-frequency content within the bandwidth of the actuator.

**Section S14. Flying-climbing transition experiments of the robot**

The flight trajectory and body attitude of the robot were digitized from the coordinates of five marker points on the robot using the DLTdv digitization tool (*62*). The coordinates of the CoM of the robot were determined to analyze the flight trajectory. The pilot controlled the entire experiment remotely. At least two cameras could see the markers throughout the flight for a full analysis, and the flight kinematics of the robot were tracked. We report the results of seven flights. The pilot determined the attitude set point through the RC stick of the remote control. The test sequences included hovering (zero pitch or roll commands), flying–climbing transition (full pitch and roll commands), and returning to hover (zero pitch or roll commands).

The optical motion tracking system was using to track the position and orientation of the robot through the reflective markers equipped on the robot, as shown in Figs. S4 and S5. To ensure effective tracking in any situation (even if the robot body is inverted), five infrared reflective points (OptiTrack, Inc.) approximately 7 mm in diameter, each weighing approximately 0.2 g, were used as marker points. One of the markers was placed on the robot tail, and the other four were placed on the plane frame of the fuselage base. The tracking marker points represent an additional payload weight of 1 g. Despite the inevitable impact on the dynamics of the robot (lower agility), performance was not significantly degraded.

**Section S15. Smooth ground takeoff and landing**

The high-speed video for the ground takeoff and landing experiments was shot at 125 fps with a shutter speed of 4000 fps (1/4000s), covering a tracking volume of approximately 1.5 m × 1.5 m × 2 m. The ground takeoff and landing experiments demonstrate the unassisted takeoff, hovering, and yaw movement capabilities of the aerial–wall robot, as shown in Fig. S6 and movie S4. In flight tests, the differences in weight and hybrid drive forces may mean that the gains that were obtained from the pre-tested force/moment system tests are too high or too low, which can affect flight stability. For this reason, several flight tests were carried out to obtain the appropriate control gain. In this experiment, we demonstrate that a hybrid aerial–wall robot with integrated rotor and flapping wing can generate enough thrust and control torque to take off, hover, circle around, and land on the ground with the support of onboard electronics and power supplies (Fig. S6 and movie S4). Our free-flying experiments demonstrate the stability and control of the robot.

The same experiment was repeated several times in the motion capture area to analyze the robot’s motion. Note that the motion capture system was not used for feedback control. Instead, it was only used to record the position and attitude of the robot. Figure S5 shows a series of 3D visualizations of motion sequences and trajectory tracking data. Figure S5A is a composite image of the free flight experiment of the aerial–wall robot, and Fig. S6B is its 3D flight trajectory. Figure S5C shows the three coordinate axis displacement data and the three attitude angle data over time in the visualization area of the robot. The robot starts from a stationary position on the ground with both the rotors and flapping wings idle (Fig. S6A(i), t = 0.0 s). The robot then simultaneously activates the rotors and flapping wings to take off from the ground, using the rotors to balance the body. After the robot reaches its highest point, it descends slightly to a certain height and then hovers (Fig. S6A(ii), t = 1.5 s). It stays hovering for approximately 1.6 s before gradually landing on the ground (Fig. S6A(ii), t = 3.6 s).

For the Z-axis, we note that the robot flies upward at a uniform speed after accelerating takeoff, and it takes approximately 1.3 s to reach an altitude of approximately 1 m. After reaching the highest point, it decelerates, accelerates in the opposite direction, and then lands at the same speed at a position (Fig. S6, t = 1.5 s) and hovers for 1.6 s. Velocity is not generated in the Z-axis direction during hovering, after which it lands on the ground at the same acceleration. This is mainly due to the linear relationship between the thrust generated by the flapping/rotor hybrid power and the control signal. The X- and Y-axes can be said to be slightly symmetrical and the robot drifts slightly along the Y-axis. Because we added the yaw control to the head rotor to demonstrate the yaw movement ability of the aerial–wall robot, the robot rises in a spiral trajectory (Fig. S6B). The forward direction plotted in Fig. S6B shows the robot moving in almost the same direction as it climbs, although it undergoes a slight rotation about its body axis (the Z-axis).

Tiny changes in the roll and pitch angles (near the 0° criterion) indicate that the attitude of the robot remains stable and upright during vertical flight. This may be the result of the correct operation of the feedback control. The yaw angle has a small change from the 0° criterion within the first 1 s, before a slight drift around the Z-axis. The yaw angle offset from the initial condition is shown during hovering. This occurs because the initial yaw moment was not fully adjusted and the control gain of the yaw was not fully determined. However, the yaw motion had no significant effect on the upright stability of the robot. Flight tests manifest that the robot could hover and circle over a ground radius of 0.15 m.

The controller of the robot used the same nonlinear flight controller as in the previous experiment because the controller can track arbitrary body attitude direction by controlling the distributed rotors. The takeoff, hovering, circling, and landing were decoupled into two control problems: upward and downward acceleration/deceleration control and steering angle control. The steering is achieved by rotating around the longitudinal axis of the fuselage through the yaw control of the vector rotor during circling around. Therefore, the yaw attitude angle changes greatly during hovering (Fig. S6C).

The upward and downward acceleration/deceleration changes in the throttle control signals are achieved by a change in rotor rotating speed without adjusting body pitch. When the robot is in a steady state, the control distribution controller of the front and rear rotors applies a torque to counteract the moment generated by the flapping wings to maintain the balance in that state. This method ignores the transient process of turning the rotor to an inclined position, during which a reverse force is generated, thus limiting the control bandwidth. However, this controller is sufficient for direct manual control. In the composite image in Fig. S6A, the robot is shown in at the initial moment of acceleration, final moment of deceleration, and the steering moment during hovering, demonstrating the stable flight.

The free flight experiments validate that active feedback is essential for the stability of the robot during flight. The robot can successfully achieve stable vertical flight and hover using the Stabilize mode. However, the angular velocity and angular acceleration curves of Fig. S6D(ii) and Fig. S6E(ii) show that the robot experienced slight oscillations during pitch and roll movements, which caused it to wander out of sight. These oscillations may be asymmetric control moments caused by slight imperfect control gains. In addition, the flapping frequency in the hybrid power is not controlled by feedback. Instead, the flapping frequency is manually controlled by the power throttle RC stick. As a result, unstable flapping frequencies during flight may have had an influence on the force generation, resulting in asymmetric stability correction moments. By further combining angular velocity and attitude feedback control, the stability of the robot can be improved.

The robot is controlled by one servo motor and four motor regulators for attitude control in the absence of a tail. A series of experimental results showed that the aerial–wall robot can generate the ideal rolling control moment by changing the wing flapping frequency. Pitch control torque can be achieved using the differential rotating speed of the front and rear rotors. The yaw control torque is generated by the rotor of the servo motor rotating part. The robot uses cascade PID controllers and an IMU to provide pitch, roll, and yaw rates, and can ultimately successfully perform stable vertical flight and yaw movements as well as movements with very little variation in pitch and roll.

**Section S16. Data processing and analysis**

In the attitude torque test, we used pulse signals to represent the number of trials before the beginning of each trial. We used the (estimated) body pitch and roll, the two signals contained in both datasets, and prefiltered them using filters (both datasets are the same). Because the sensor sampling frequency is 1 Hz, we used spline interpolation for the attitude command corresponding to the PWM wave in the onboard datasets (interp1 (..., ..., 'spline') in the MATLAB function), and interpolated it to 1 kHz. The time difference was calculated from the relative displacement of the signal, giving the maximum cross-correlation (xcorr(...) in the MATLAB function). The average of the roll and pitch was used for the synchronized datasets.

From the 3D marked positions, we calculated the position and orientation of the fuselage, represented by their three Euler angles (Fig. S5), and analyze the flight trajectory and body attitude kinematics of the robot. To determine the digital body attitude (pitch angle , roll angle , and yaw angle ), we tracked the five points (1 to 5 in Fig. S4B). These points are at the end of the fuselage of the longitudinal axis (point 1) and at the two ends of a horizontal line in the plane of the body’s CoM (points 4 and 5). In addition, points 2 and 3 are in the same horizontal plane as points 4 and 5, and these four points are arranged such that they divide the back half of the plane into three 60° sections. The centroid of the body is located at the intersection between the center points of markers 4 and 5 vertically upward and the vertical axis (Fig. S5B). Their 3D coordinates (with 95% confidence intervals) are obtained from the coordinate information of the customized calibration rod (length 25 cm × width 20 cm × height 20 cm). The flight trajectory is analyzed from the coordinates of the CoM.

We define the fixed global coordinate system Qg = {Xg, Yg, Zg}(Fig. S5A) and the fixed robot body coordinate system Qb = {Xb, Yb, Zb} (Fig. S5B). In the Qg system, the XgYg plane corresponds to the horizontal plane and the Zg-axis is perpendicular to the horizontal plane. In the Qb system, the Xb-axis points to the right of the flapping-wing base (points 4 to 5), the Yb-axis points to the head of the robot, and the Zb-axis is the cross product of the Xb- and Yb-axes. We define the Euler angles according to the rotation sequence of pitch–roll–yaw, following the standard aerospace equation (*63*). The origin of the body axis is at the CoM, and the body CoM velocity and acceleration are calculated by numerical differentiation of the position vector (on the fixed ground axis). These data are transformed using Euler angles to determine the velocity, acceleration, angular velocity, and angular acceleration of the robot in the body coordinate system.

Owing to occasional trace loss, outliers or short segments of blank space (up to 10 frames) exist in the data. They are interpolated with linear interpolation (interp1(..., ..., 'linear') in the MATLAB function). Experiments with longer abnormal segments or gaps are discarded. The frame when the robot enters the sight is selected as the origin of each video, and the frame when the robot is about to leave the sight after the transition is completed is selected as the destination.

The labeled data are used to construct a reference frame related to the robot body (Fig. S5). These reference frames (Qg and Qb) are related to each other using a rotation matrix. The rotation matrix is a 3 × 3 matrix used to convert the components of a vector between two different reference frames considering the attitude angle around the axis of the body for construction. The vector construction is simple when the position and size of the marker in the body coordinate system are known, whereas in the global coordinate system, the vector is determined from the marker data. We observe that the rotation matrix linking the global and body coordinate systems is simply constructed from the unit vectors of the body frame in the global coordinate system (Equation (33)).

|  | (33) |
| --- | --- |

For that, we use Z-Y-X Euler angles. These angles represent three rotation sequences of an object rotating between the initial and final directions (*64*). The first rotation rotates the coordinate system positively by an angle about the original Xb-axis. The second rotation rotates it by an angle around the new Yb-axis. The last rotation rotates it by an angle about the final new Zb-axis. Each of these rotations can be individually written as a rotation about a particular axis (Equation (33)), and when combined, we obtain the full rotation matrix as

|  | (34) |
| --- | --- |

In this equation, we represent sin and cos in terms of s and c. Hence, we can briefly determine the Euler angles of the rotation matrix as

|  | (35) |
| --- | --- |

We define the general orientation of the aerial–wall robot using the concepts of the body coordinate system and Euler angles. The Euler angles associated with this coordinate system have specific designations and implications. The first rotation is the pitch angle of the robot, which represents the amount of inclination of the body with respect to the global coordinate system’s XgYg plane. The second rotation is the roll angle of the robot, which is the amount the robot has rotated about the Yb-axis of the body. The third rotation is the yaw angle, which represents the orientation the robot is facing.

We need to pay attention to the gimbal lock when choosing the Euler angles, which for our definition of Euler angles is the attitude of the robot when it is at *θ* = ±90°. In the flying–climbing transition experiments, the robot’s body will approach this limit during adhesion and wall climbing. There are two solutions when converting rotation matrices to Euler angles. When recording these two solutions for any given time step i, we can simply compare them with the values for time step i − 1 and choose the values that are closer with respect to the three attitude angles. Because of the continuity of rotation, the combination that is closer to the value of the previous moment is the correct solution. The result describes the flight trajectory of the robot using the Euler angles.

**Figures:**


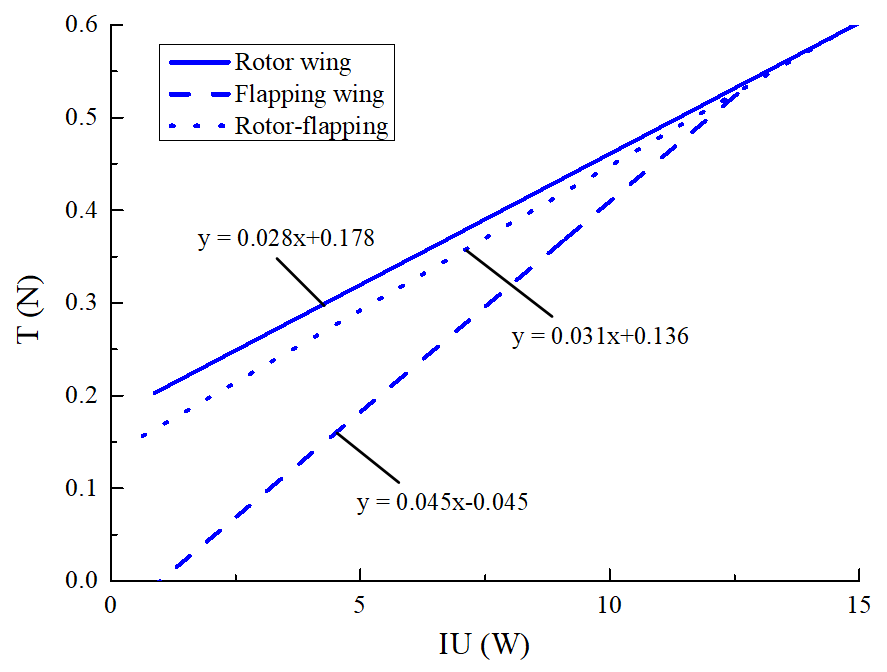


**Fig. S1. Comparison by thrust generated (T) as a function of input electrical power (IU).**


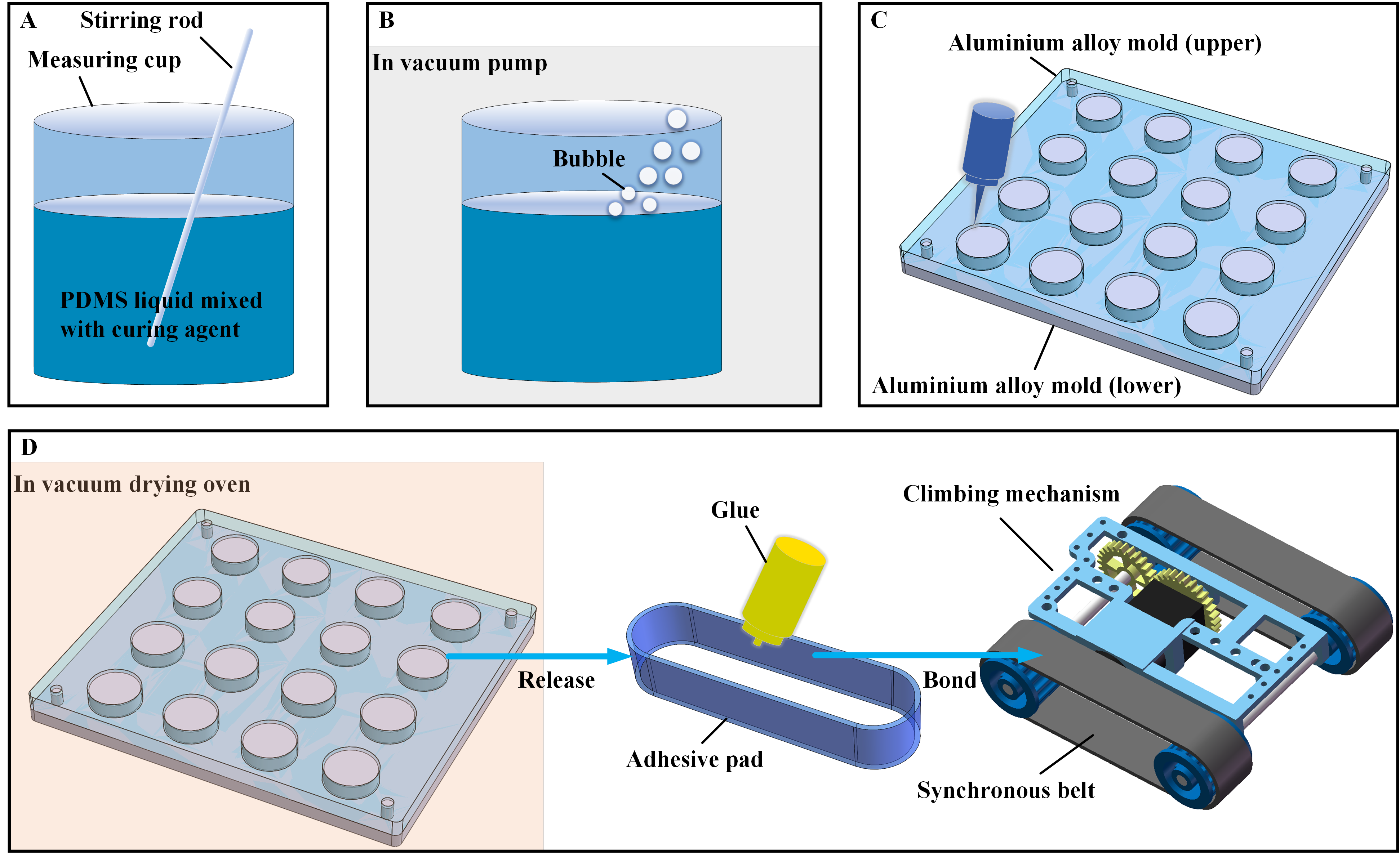


**Fig. S2. Fabrication of the adhesive pad.** (**A**) Mixing the PDMS liquid (Sylgard 184, Dow Corning) in a ratio of 20:1 with a curing agent and stirring for 3–5 minutes. (**B**) Putting the mixture into a vacuum pump for 30 minutes with a negative pressure of 0.8 to pull the bubbles from the liquids. (**C**) Casting the degassed mixture on a special aluminum alloy mold. We used a 355 nm diode-pumped solid-state laser micromachining system (DC150H-355, Photonics Industries International Inc., NY, USA) to machine the upper and lower aluminum alloy molds. (**D**) The mold was put into a vacuum drying oven for 10 minutes in static with negative pressure at 2.5, and then kept at a high temperature and low pressure for 2 hours, and cooled to room temperature naturally. The upper mold was removed and the adhesive material was demolded in water without physical or chemical damage. Finally, we glued the inner side of the adhesive pads with a silicone rubber adhesive (Sil-Poxy, Smooth-on, Inc, Easton Pa, USA) and used it to cover the synchronous belts.


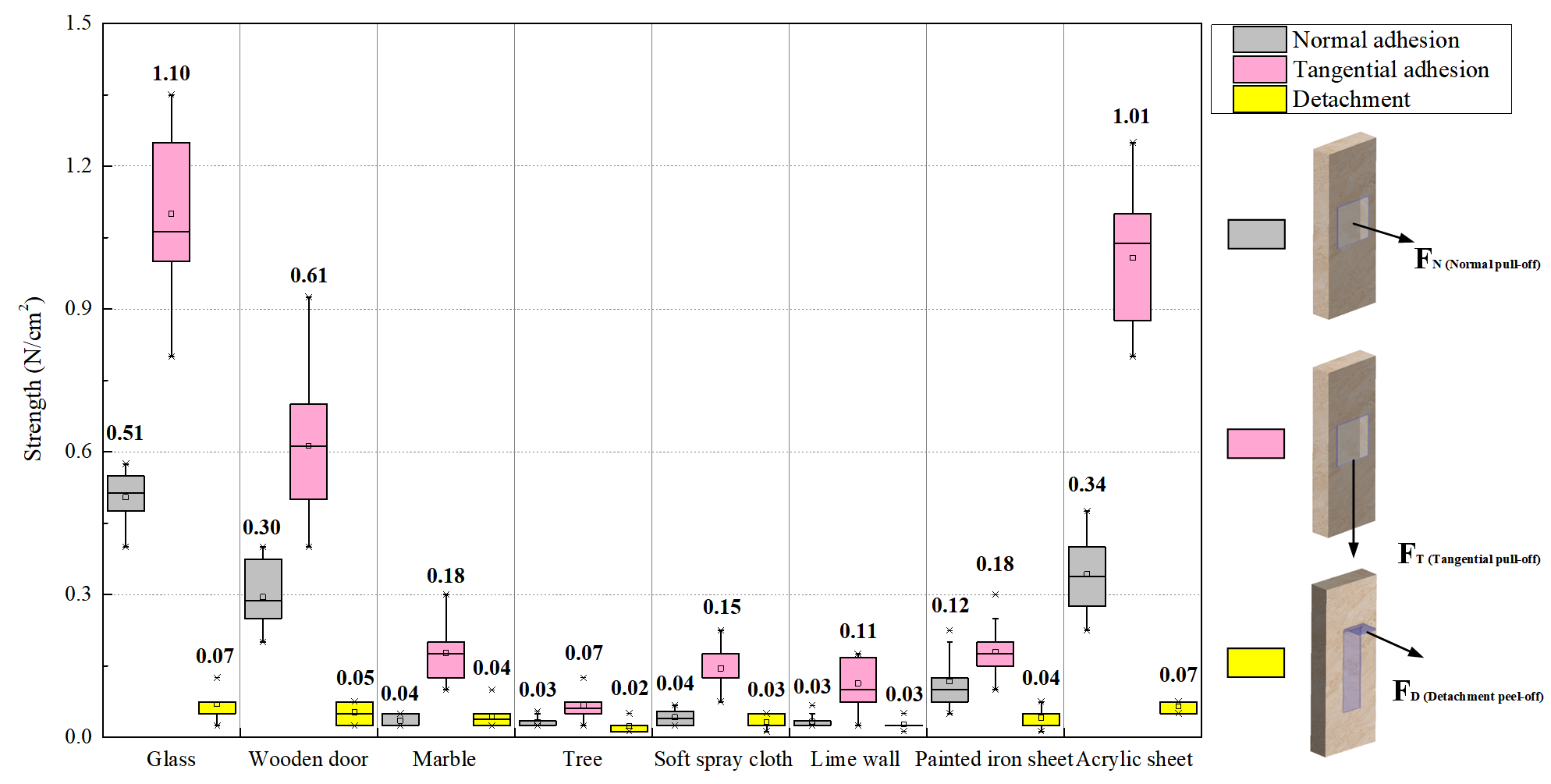


**Fig. S3. Boxplots of the measured adhesion performances of PDMS adhesive samples patches in normal pulling, tangential pulling, and peeling directions on different surfaces.** * P < 0.05, ** P < 0.01, *** P < 0.001; only the significant comparisons between different surfaces are displayed (N = 10). The numbers above the box represent the average strength values.


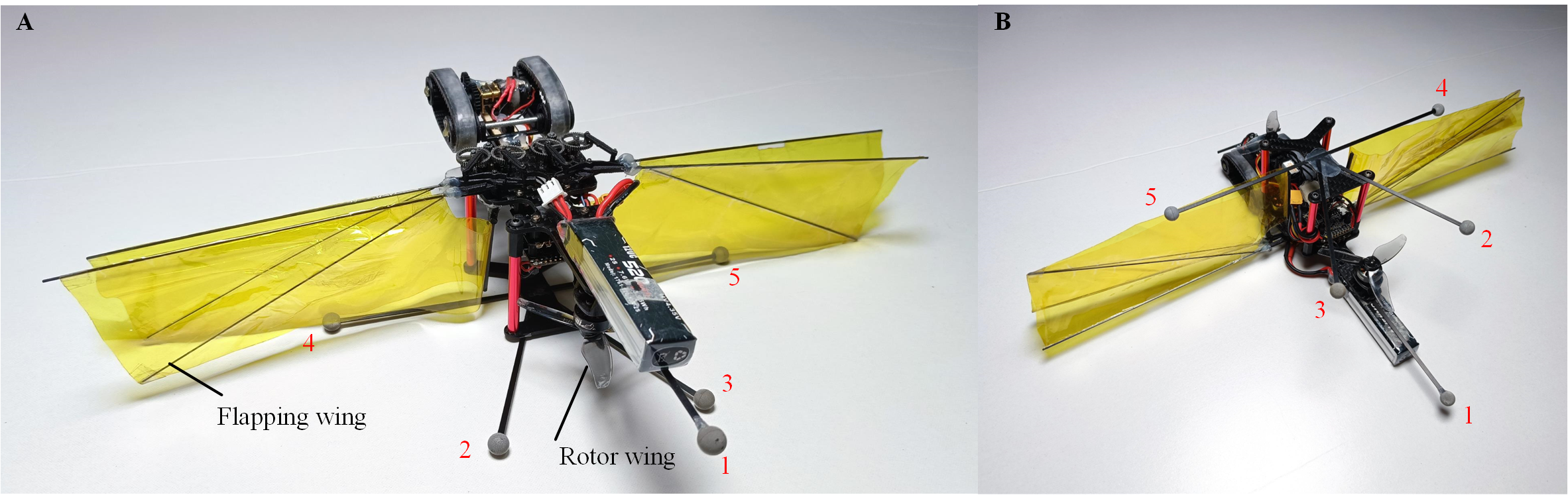


**Fig. S4. Robot with reflective markers for the motion tracking system.** (**A**) Three approximately spherical markers from the side view of the robot. (**B**) Two additional collinear circular markers (4 and 5) were used for better processing the Euler angle (top view). The weight of the five markers (OptiTrack, Inc.) is negligible (0.2 g).





**Fig. S5. Experimental setup and body coordinates.** (**A**) Experimental setup for the transition between flying and climbing during robot flight recorded by four synchronized high-speed cameras. A fixed global coordinate system Qg = {Xg, Yg, Zg} was defined to determine the flight path of the robot. (**B**) Definition of the body coordinates of the robot Qb = {Xb, Yb, Zb}.


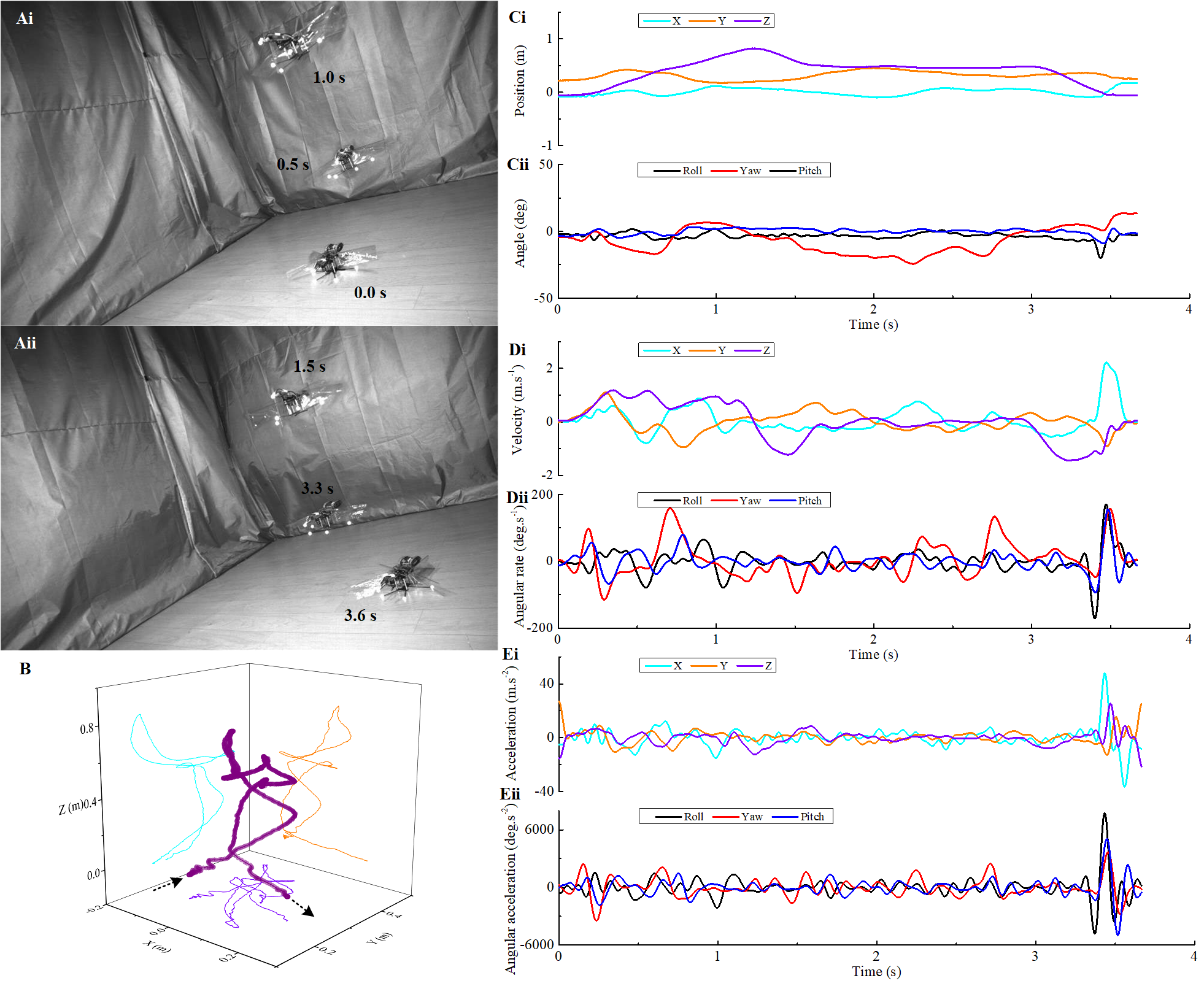


**Fig. S6. Takeoff, hovering, and landing flight tests.** (**A**) Composite images of (i) taking off and (ii) landing. (**B**) flight trajectory. (**C**) Position versus time (i) and body attitude angles versus time (ii). (**D**) Velocity versus time (i) and body angular rates versus time (ii). (**E**) Acceleration versus time (i) and body angular accelerations versus time (ii). Black dotted lines with arrows in (B) represent the direction of motion.

**Tables:**

**Table S1. List of robot components and their weights.**

| **Component** | **Number** | **Unite Weight (g)** |
| --- | --- | --- |
| Robot body structure | 1 | 12.8 |
| Flapping wing | 2 | 0.92 |
| Kakute F7 mini V2 Autopilot | 1 | 4 |
| AC900 Receiver | 1 | 0.91 |
| ALIENMODEL HV_520mah Battery | 1 | 30.48 |
| HP03SE Coreless Motor (flapping) | 2 | 3.2 |
| GA12-N20 Micro Gear Motor (climbing) | 1 | 9.5 |
| Micro Brushed ESC (climbing) | 1 | 0.37 |
| T-MOTOR F1103 Brushless motor (rotor) | 2 | 5.15 |
| HAKRC15A ESC | 1 | 4.5 |
| BLUEARROW D03018MG Servo | 1 | 3.7 |
| Flapping mechanism | 2 | 3.58 |
| GEMFAN2512_3 Propeller | 2 | 0.8 |
| Climbing mechanism | 1 | 8 |
| Climber belt & adhesive material | 2 | 4.56 |
| Wiring, glue & other |  | 24.7 |
| Total weight |  | 135.38 |

**Table S2. Flying-climbing transition trials of aerial–wall robots on different surfaces.**

| **Different surfaces** | **Number of trials (n)** | **Statistics of success (n)** | **Percentage (%)** |
| --- | --- | --- | --- |
| Glass | 10 | 8 | 80 |
| Wooden door | 10 | 7 | 70 |
| Marble | 10 | 8 | 80 |
| Tree | 10 | 3 | 30 |
| Soft spray cloth | 10 | 5 | 50 |
| Lime wall | 10 | 3 | 30 |
| Painted iron sheet | 10 | 6 | 60 |
| Acrylic sheet | 20 | 17 | 85 |

**Table S3. Parameters of the robot performance.**

| **Parameter** | | | **Value** |
| --- | --- | --- | --- |
| Flying | Flapping wing | Flapping amplitude | 90° |
|  |  | Wing span | 33 cm |
|  |  | Hover flapping frequency | 15−20 Hz |
|  | Rotor wing | Propeller dimensions | 1.9 inch |
|  | Flight performance | Flight endurance | > 4 min |
|  |  | Sustainable flight distance | > 1 km |
|  |  | Flight speed | 6.8 m/s |
| Climbing | Climbing mechanism | Climbing speed | 6 cm/s |
